# Strategic behaviour and decision making in competitive hospital markets: an experimental investigation

**DOI:** 10.1007/s10754-024-09366-3

**Published:** 2024-03-15

**Authors:** Johann Han, Nadja Kairies-Schwarz, Markus Vomhof

**Affiliations:** 1 Faculty of Economics and Business Administration, University of Duisburg-Essen, Essen, Germany; 2 CINCH (Competent in Competition and Health), Berliner Platz 6-8, 45127 Essen, Germany; 3 Institute for Health Services Research and Health Economics, Centre for Health and Society, Medical Faculty and University Hospital Düsseldorf, Heinrich-Heine-University Düsseldorf, Düsseldorf, Germany; 4 Institute for Health Services Research and Health Economics, German Diabetes Center, Leibniz Center for Diabetes Research at Heinrich-Heine-University Düsseldorf, Düsseldorf, Germany

**Keywords:** Quality competition, Hospital markets, Team decisions, Altruism, Laboratory experiment, C92, D03, D43, D64, I11, L13

## Abstract

We investigate quality provision and the occurrence of strategic behaviour in competitive hospital markets where providers are assumed to be semi-altruistic towards patients. For this, we employ a laboratory experiment with a hospital market framing. Subjects decide on the quality levels for one of three competing hospitals respectively. We vary the organizational aspect of whether quality decisions within hospitals are made by individuals or teams. Realized monetary patient benefits go to real patients outside the lab. In both settings, we find that degrees of cooperation quickly converge towards negative values, implying absence of collusion and patient centred or competitive quality choices. Moreover, hospitals treat quality as a strategic complement and adjust their quality choice in the same direction as their competitors. The response magnitude for team markets is weaker; this is driven by non-cooperative or altruistic teams, which tend to set levels of quality that are strategically independent.

**Supplementary Information:**

The online version contains supplementary material available at 10.1007/s10754-024-09366-3.

## Introduction

In hospital markets where prices are regulated and patients can choose hospitals freely, quality has become a crucial strategic variable. There is a rich set of literature studying the effects of competition on quality (see, for example, Gaynor & Town, 2012; Gaynor et al., 2015 for surveys). However, little is known about the strategic considerations behind hospitals’ quality provision behaviour and whether the way it is materialized within hospitals has an effect on the quality provided.^1^ This is relevant in healthcare markets, since providers who decide on the quality of care on either an individual or team level are typically assumed to be semi-altruistic (Chalkley & Malcomson, 1998; Chone & Ma, 2011; Ellis & McGuire, 1986, 1990; Jack, 2005; Liu & Ma, 2013). Several laboratory experiments have identified such altruism in medical provision behaviour for individual decisions (Brosig-Koch et al., 2016, 2017a; Godager & Wiesen, 2013). Hence, when heterogeneously semi-altruistic providers are competing, the outcome might be different from classic market experiments with profit maximizing agents.^2^

Using a theoretical model, Brekke et al. (2017) find that the strategic nature of quality competition crucially depends on the hospitals’ degree of altruism and the interaction between quality and cost-containment incentives. Without room for cost containment, they show that quality is a strategic complement if altruism is relatively low compared to the cost-substitutability between quality and output; if altruism is relatively high, quality can also be a substitute. In contrast to the theoretical predictions, the empirical literature on hospitals’ strategic interactions considering the strategic nature of quality in hospital markets is rather small and not straightforward—that is, if at all they find positive significant associations between rivals’ quality choices. Gravelle et al. (2014) investigate whether a hospital’s quality depends on the quality of rival hospitals using data from the English National Health Service and find mixed results: only some quality indicators are positively associated and are thus complements. Yet, they do not find evidence for any negative association. In a follow-up study, Longo et al. (2017) investigate a longer time horizon and only find significant positive associations for one out of eight quality indicators. Guccio and Lisi (2016) use data from Italian hospitals and investigate the strategic nature of caesarean section rates, showing that a hospital’s provision behaviour is strongly affected by the behaviour of the other hospitals within one region.

However, data from the field might be prone to certain challenges, making a controlled analysis difficult. For instance, a necessary assumption for provider coordination and demand effects is that quality is observable, which is not necessarily the case. While reforms including policies targeting increased transparency by publicizing physician quality information have gained popularity (Dranove & Jin, 2010), other aspects, such as the multidimensionality of quality—often in combination with subjective perceptions of overall quality, as well as neglecting quality reports entirely—lead to a lack of transparency in the field. This makes coordination much more difficult.

Even if quality responses were perfectly observable from field data, it would be difficult to infer whether providers directly reacted to their competitors’ quality choices or whether their quality choice was an outcome of the decision process within the hospital and thus derived from adhering to a certain social norm or treatment style. Hence, in order to understand hospitals’ quality responses, one should also account for the organizational structure of the decision processes that leads to the quality provided. Typically, the decision on strategic orientation is not just made by an individual but in organized teams like management boards, including, for example, the head of the hospital, the chief financial officer, and the senior physician. Each of them might weigh hospital performance and thus quality differently. Then, the unitary player assumption that is common in the industrial organization literature might be less reasonable if the involved stakeholders put different weights on profit and patient centredness.^3^

The objective of this paper is to investigate how competing and semi-altruistic hospitals provide quality when decisions are made on either the individual or team level. For this, we use data from the first part of a controlled laboratory experiment on individual and team hospital markets conducted by Han et al. (2017). We conducted additional experimental sessions to increase the number of observations for team markets. While Han et al. (2017) compare parts 1 and 2 of the experiment and focus on the analysis of a merger’s total effects on quality provision, we focus on hospitals’ strategic interactions when varying the way decisions are made (i.e., whether at an individual or team level). In the experiment, participants take on the role of a decision maker in a hospital and have to choose a quality level for their respective hospitals in 15 consecutive periods. The market consists of three hospitals that compete for patients by their quality choice in a repeated Salop oligopoly game. We also implement an experimental condition with a team decision process. Here, hospital boards of three members have to agree on a quality level through a simple majority rule. Decisions affect real patients outside the laboratory, since the generated patient utility is monetized and transferred to a charity that provides medical care to patients without access to formal healthcare.

Our paper contributes to the growing literature on health economic experiments on provider behaviour (Hennig-Schmidt et al., 2011; Hennig-Schmidt & Wiesen, 2014; Green, 2014; Brosig-Koch et al., 2016, 2017a, 2017b, 2020; Lagarde & Blaauw, 2017; Kesternich et al., 2015; Di Guida et al., 2019; Martinsson & Persson, 2019; Reif et al., 2020; Kairies-Schwarz & Soucek, 2020). While there is some research investigating competitive markets with semi-altruistic healthcare providers, showing that semi-altruism yields better patient outcomes than expected for profit maximizing agents (Brosig-Koch et al., 2017b; Han et al., 2017), there is no evidence on strategic behaviour in hospital markets with quality competition that includes a comparison of individual and team decisions.^4^ Our paper also contributes to two strands of literature on classic market experiments. The first strand investigates tacit collusion in repeated Bertrand and Cournot markets (Potters & Suetens, ), showing that it is more difficult to establish collusion when strategic variables are substitutes. The second one analyses team decisions in market games, suggesting that teams are generally closer to the theoretical predictions of the game than individuals are (see, for example, Bornstein et al., 2008; Kugler et al., 2012 for surveys).^5^ However, our setting is unique because, given the externalities for patients, semi-altruism is a plausible assumption in these markets, and the theoretical benchmarks of the game with profit maximizing agents might be less relevant.

Our main results can be summarized as follows. We show that individual and team markets start at slightly positive degrees of cooperation. However, they quickly converge towards negative values, indicating patient-centred behaviour. We also show that initial quality is significantly more homogeneous in team markets than in individual ones. This might be explained by the preceding team decision process, with the majority voting, filtering out extreme quality choices from the beginning. Towards the final periods, both hospital markets coordinate at levels between the competitive and optimal patient outcome. We also consider strategic quality provision behaviour and find that hospitals led by an individual treat quality as a strategic complement and adjust their quality choice in the same direction as their competitors. This also holds for hospitals led by teams. However, their response magnitude is weaker. This is driven by non-cooperative, or altruistic, team markets, which do not seem to be responsive to changes in their rivals’ qualities but tend to set quality independently.

The rest of the paper proceeds as follows. Section "Experimental design and theoretical framework" outlines the experimental design and theoretical framework. Section “Results” presents the results, and Section “Conclusion” concludes the study.

## Experimental design and theoretical framework

### Experimental design and decision situation

#### Individual decisions

Our setting is similar to a classical market experiment but with a healthcare framing. Instead of firms, there are hospitals competing for patients. Participants are randomly matched into a market and are in charge of quality decisions for one of three competing hospitals. At the beginning of each period, subjects simultaneously choose the quality level $q$ that they want to provide, with $q \in \left\{ {1,2,3, \ldots ,13} \right\}$.^6^ The quality level affects patient utility—the higher the better—which determines the patients’ hospital choice and therefore market demand and hospital profits. Overall, participants play 15 periods where they decide on the quality. In line with the theoretical model of quality competition, hospitals decide on the quality of care. We assume that a minimum standard of quality is always fulfilled to ensure that a patient’s problem is solved. Any additional quality will continuously increase patient benefits. Such additional quality provision might also be reflected in (publicly available) quality indicators that make quality observable to the patient. Assume, for instance, the area of obstetrics: in the event of a premature birth, the presence of a paediatrician leads to an increased quality level. In Germany, for example, this aspect is also reflected in quality indicators.

Subjects are provided with full information about the possible consequences of their own and their competitor hospitals’ quality decisions with respect to profits and patient benefit. A profit and patient benefit table is handed out with the instructions (see Appendix A.1.1 and A.2.1 for the instructions translated into English, as well as the original ones in German), and a calculator is implemented in the computer program where subjects can simulate different quality combinations in the market with the respective outcomes (see Appendix A.2.2 for translated screenshots of both individual and team market conditions).

Patient utility is measured as the total utility derived by the unit mass of patients in the market from the quality provided by the three hospitals (more detail in Section "Behavioural benchmarks and hypotheses"). Subjects can use the implemented calculator to calculate their own contribution and the contribution by the other hospitals. There are no real patients in the lab. To create a more realistic decision situation that allows for altruism towards patients, we implemented a transfer of the monetary equivalent of quality choices, similar to Eckel and Grossman (1996), Hennig-Schmidt et al. (2011), and Brosig-Koch et al., (2016, 2017a, 2017b). Participants in the experiment knew that the higher the level of quality provided, the more money would go to a charity granting access to healthcare to uninsured patients in Germany who would not be treated otherwise.

#### Team decisions

Our main ceteris paribus variation is the way decisions are made.^7^ In addition to individual decisions, we have a team condition where subjects are matched in teams of three (throughout the experiment, the same team members constitute a team) and form a hospital board that makes decisions about hospital quality. The match remains until the end of the game. With three subjects on each hospital board, nine players form a market. Hospital quality level $q$ is determined by a majority vote of the hospital board members. The decision mechanism is a simple majority rule, similar to Gillet et al. (2011). If at least two members propose the same quality level, it is implemented as the hospital quality level for the current period. If no majority is reached within a voting round, the same voting process is continued until a majority decision is reached. Our experimental conditions thus vary regarding whether decisions are made at the individual or team level (see Table 1).

**Table 1:** Experimental conditions overview

Conditions	Number of subjects	Number of hospitals	Number of markets^a^
Individual	213	213	71
Team	342	114	38
Total	555	327	109

^a^All observations for the individual markets and for 13 team markets are taken from Han et al. (2017). We ran further sessions to get additional 25 independent observations for team markets. To be in line with Han et al. (2017), all sessions consisted of two parts, where the first part was an individual or team competition scenario and the second part went along with a change in market concentration. In this study, we are only interested in the first part, so we can pool the observations within all individual and team conditions from Han et al. (2017). See Appendix A.1.2 for a detailed explanation on how we derived the respective sample sizes

### Behavioural benchmarks and hypotheses

In the following section, we derive behavioural benchmarks and hypotheses for our experiment. For the individual hospital markets, we refer to the theoretical framework introduced in Han et al. (2017), who investigate competitive market outcomes with and without hospital mergers. For the team hospital markets, we present the related literature and infer our hypotheses from it.

#### Individual decisions

We begin by introducing the model framework for individual quality decisions. For this, we consider a Salop model with an exogenously fixed number of three hospitals that compete in terms of treatment quality (Salop, 1979). A unit mass of patients is uniformly distributed on a circle. Patients receive medical treatment at hospitals that are equidistantly located along the circle. A patient’s utility depends on the quality level $$q_{i}$$ received in hospital *i* with $$i \in \left\{ {1,2,3} \right\}$$, as well as on the travel distance between the hospital’s location $$x_{i}$$ and the patient’s location $$z$$. The disutility from traveling is measured by $$t > 0$$. Patients are fully insured, i.e., prices for treatment do not affect their utility. Furthermore, it is assumed that the ‘basic’ valuation of treatment *v* is sufficiently large to ensure that receiving treatment is always preferred to remaining untreated.^8^ Given the hospital’s location $$x_{i}$$ and the patient’s location $$z$$, the patient’s utility $$u_{{z,x_{i} }} $$ is given by:

1 $$ u_{{z,x_{i} }} = v + q_{i} - t \left| {z - x_{i} } \right| . $$

The total utility of patients being treated by the hospital i is $B_{i} = \mathop \smallint \limits_{o}^{{\hat{z}_{i}^{i + 1} }} \left( {v + q_{i} - ts} \right)ds + \mathop \smallint \limits_{o}^{{\hat{z}_{i} - 1}} \left( {v + q_{i} - ts} \right)ds,$ which determines the contribution to the patient in the following. It can be shown that hospital *i*’s demand $D_{i}$ depends on the quality choices of all three hospitals active in the market and is given by:

2 $$ D_{i} = \frac{1}{3} + \frac{{2 q_{i} - \mathop \sum \nolimits_{j \ne i} q_{j} }}{2 t}. $$

Hospitals compete for patients in terms of quality.^9^ Since prices *p* for treatment are exogenously given by a regulator and marginal costs $c$ > 0 per quality are constant, hospital *i*’s profit function can be written as:

3 $$ {\uppi }_{i} = \left( {p - c q_{i} } \right) D_{i} . $$

The three competing hospitals simultaneously choose their quality level in order to maximize their profit function as stated in Eq. (3).^10^ If we take the first-order condition (FOC), we can solve for the best-quality-response function for each hospital:

4 $$ q_{i} \left( {q_{j} } \right) = \frac{1}{2}\frac{p}{c} + \frac{{\mathop \sum \nolimits_{j \ne i} q_{j} }}{4} - \frac{t}{6} . $$

The corresponding symmetric Nash equilibrium quality $$q_{i}^{*} $$ and profit level $${\uppi }_{i}^{*}$$ of hospital *i* is given by:

5 $$ q_{i}^{*} = \frac{p}{c} - \frac{t}{3} {\text{and}} {\uppi }_{i}^{*} = \frac{c t}{9} . $$

To derive theoretical benchmarks and our behavioural expectations for the strategic nature of quality provision within our experimental design, we now introduce our experimental parametrization of the formal model. We chose our parameters in a way that satisfies the participation constraint for patients—that is, demanding treatment even under the hospital’s minimal quality provision of 1 ($v = 5$) and that patients do not travel beyond one of their neighbouring hospitals to receive a treatment ($t = 36$). Regulated prices are set at $p = 44$ and treatment costs at $c = 2$. Subjects could choose a quality level $q_{i} \in \left\{ {1,2,3, \ldots ,13} \right\}$.^11^

Based on this parametrization, we introduce some relevant benchmarks (see Table 2). The symmetric Nash equilibrium is reached at a joint quality level of 10, where each hospital makes a profit of 8 Taler^12^ while the total utility of patients (contribution to patients) is 4 Taler. The patient optimum is at a quality level of 13, with a lower per hospital profit of 6 Taler and a higher contribution to the patient of 5 Taler. When all hospitals choose the minimum quality level of 1, the joint profit maximum (JPM) is reached. Every hospital gets a profit of 14 Taler in this period, and 1 Taler goes to the patient population. The benchmark defect refers to defecting from this collusive JPM. The optimal defection choice for purely profit maximizing providers is a quality level of 5 or 6,^13^ both leading to profits of 15.11 Taler (a full profit and patient utility table can be found in Appendix A.1).

**Table 2:** Theoretical benchmarks

	Quality level	Profit	Patient utility	Degree of cooperation
Nash equilibrium	10	8	4	0
Patient optimum	13	6	5	-0.33
JPM	1	14	1	1
Defect from JPM	5 or 6	15.11	2.67 or 3.19	

Profit and patient utility for JPM, Nash equilibrium, and patient optimum are in symmetric scenarios where all providers choose the same level of quality. For Defect from JPM, profit and patient utility only apply to the defector while the other providers are still choosing a quality level of 1

Next, we introduce a measure for the degree of cooperation within a market. Following Suetens and Potters (2007) and Potters and Suetens (2009), we translate these quality benchmarks into degrees of cooperation. This gives us a straightforward indication about how collusive a healthcare market actually is. The degree of cooperation for healthcare market $k$ in period $t$ is calculated by:

6 $$ \rho_{kt} = \frac{{Avg. Market Quality_{kt} - Quality^{Nash} }}{{Quality^{JPM} - Quality^{Nash} }}. $$

Given our parametrization, it holds that $\rho_{kt} = 0$ for average quality choices at the non-cooperative Nash equilibrium of 10. The JPM would result in $\rho_{kt} = 1$, while a uniform quality choice at the patient optimum would result in $\rho_{kt} = - 0.33$.

So far, our theoretical framework assumes pure profit-maximizing hospitals. However, when assuming semi-altruistic hospitals, similar to Brekke et al., (2011, 2017), a hospital $i$’s objection function can be written as:

7 $$ {\Omega }_{i} = \left( {p - c q_{i} } \right) \cdot D_{i} + \alpha \cdot B_{i} $$

where $B_{i} = \mathop \smallint \limits_{o}^{{\hat{z}_{i}^{i + 1} }} \left( {v + q_{i} - ts} \right)ds + \mathop \smallint \limits_{o}^{{\hat{z}_{i} - 1}} \left( {v + q_{i} - ts} \right)ds$ is the total utility of the patients being treated in hospital $i$ and $\alpha > 0$ is a measure for the degree of altruism.^14^ The optimal Nash equilibrium quality, best response function, and strategic relationship thus depend on the degree of altruism (see Appendix A.3 for the formal model equations). The best response function allows hospitals’ quality levels to be strategic complements as well as a strategic substitutes. However, for semi-altruistic hospitals that maximize their objective functions, quality would be a strategic complement. Moreover, we show that our parametrization quality levels of semi-altruistic hospitals are higher (i.e. $q_{i\alpha }^{*} > q_{i}^{*} )$ and profits are lower compared to pure profit-maximizing hospitals (i.e. ${\uppi }_{i\alpha }^{*} < {\uppi }_{i}^{*} )$.

The focus of this paper is to better understand strategic behaviour within hospital markets. Therefore, we next translate our expectations to the degree of cooperation. Given our parametrization, the degree of cooperation is $\rho_{kt} = - 0.33$ for uniform quality choices at the patient utility optimum of 13 (see Table 2). Hence, in contrast to profit-maximizing providers, for which the Nash equilibrium predicts a symmetric quality choice of 10 that translates into a degree of cooperation of 0, markets with semi-altruistic providers should supply higher quality levels and have a negative degree of cooperation. In Potters and Suetens (2009), negative values of the degree of cooperation $\rho_{kt}$ are interpreted as high competitiveness beyond the Nash equilibrium. In our setting, however, negative values also correspond to average quality choices towards the patient utility optimum.^15^

##### Hypothesis 1

(*Degree of Cooperation*) In a market with semi-altruistic hospitals, we expect a negative degree of cooperation.

##### Hypothesis 2

(*Strategic Nature*) In markets with semi-altruistic hospitals, we expect that hospitals’ quality levels are strategic complements.

#### Team decisions

Next, we derive hypotheses for team decisions based on the existing empirical and experimental evidence.^16^ Comparing individual decisions with team decisions relates to the discussion about the ‘unitary player assumption’ and whether team behaviour and individual behaviour are equivalent. This might be even more important in healthcare markets, as providers’ preferences concerning profits and patient benefit might be heterogeneous in the internal decision-making process. While health economic experiments investigating healthcare provider behaviour have observed heterogeneity in the altruism of providers (Godager & Wiesen, 2013; Brosig-Koch et al., 2016, 2017a), little is known about team decisions with potentially heterogeneously altruistic healthcare providers.

There is a vast literature on group behaviour in different economic experiments (see, for example, Engel, 2010 for a survey).^17^ Some studies investigate group behaviour in market competition experiments with homogenous goods. Bornstein et al. (2008), for instance, find that teams have a harder time establishing tacit collusion than individuals in Bertrand duopolies. Bornstein and Gneezy (2002) also find that teams converge to the competitive solution quicker. Raab and Schipper (2009), in contrast, do not find differences for three firm Cournot oligopolies. In our context of quality competition between groups, there is no evidence yet on how altruistic motives interact with the strategic nature of quality.

For team decisions, the voting rule—here, the majority voting rule—might also impact decisions. For example, Gillet et al. (2011) showed that different decision rules—either by single persons who decide for their team members (‘CEOs’) or by a consensus or majority voting rule in a Bertrand pricing game—affect asking prices. In particular, for the comparable situation in which no cartel is formed, they find that prices are higher with a CEO and majority voting rule compared to the consensus rule and individual treatment. For profit-maximizing markets, we might expect lower quality levels and hence a higher degree of cooperation with the majority voting rule compared to individual decision making. However, there is no evidence for semi-altruistic teams applying the majority voting rule.

##### Hypothesis 3

(*Individual vs. Teams—Degree of Cooperation*) Given the existing empirical evidence, we expect a higher degree of cooperation for profit-maximizing agents in team decisions with the majority voting rule compared to individual decisions. The effect for semi-altruistic hospitals is ambiguous.

##### Hypothesis 4

(*Individual vs. Teams—Strategic Nature & Coordination Behaviour*) Given the existing empirical evidence, the effect of team decisions on coordination behaviour and the strategic nature of quality provision in markets with profit-maximizing as well as semi-altruistic hospitals is ambiguous.

### Experimental procedure

The experiment was programmed using the software z-Tree (Fischbacher, 2007) and conducted at the Essen Laboratory for Experimental Economics (elfe) at the University of Duisburg-Essen, Germany in 2015. A total of 555 students (213 in individual conditions and 342 in team conditions) was recruited from different fields of study using the recruiting system ORSEE (Greiner, 2015).^18^

Upon arrival, subjects were randomly assigned to seats in the laboratory and received written instructions for the first part of the experiment.^19^ They were informed that the experiment consisted of two consecutive parts, but they only received the instructions for the second part after having finished the first part of the experiment. After participants read the instructions, they could ask comprehension questions that were answered in private. Then, they were all asked to answer comprehension questions on their screen.^20^ The experiment did not start unless all subjects had answered the comprehension questions correctly. At the end of the experiment, subjects had to complete a short questionnaire with demographic and experiment related questions (see Appendix A.2.3). On average, a session lasted 120 min. Monetary amounts were displayed in the Taler experimental currency, with an exchange rate of 1 Taler = 0.07€ in the individual conditions and 1 Taler = 0.21€ in the team conditions. In the team conditions, the average payoff subjects received in the experiment (for part 1) was 17.20€ (7.57€), and the average contribution to patients was 8.50€ (4.58€). In the individual conditions, the average payoff per subject was 17.75€ (7.98€), and the average contribution to the patient was 8.14 € (4.37€).

## Results

### Data description

To begin, we provide the demographic information of the students participating in the experiment for the total sample, as well as by experimental condition (see Table 3). The total sample consists of 51.53% females. Participants were on average 23.96 years old and had studied about six semesters. Of the participants, 35.86% were economics students and 4.86% of the participants were medical students.^21^ The vast majority of participants were unmarried (92.97%).

**Table 3:** Demographic Data of the Sample and by Experimental Condition

	Total (N = 555)	Missing	Individual markets (N = 213)	Missing	Team markets (N = 342)	Missing	*p* value
Gender (1 = female), N (%)	286 (51.53%)	0	110 (51.64%)	0	176 (51.46%)	0	0.967^a^
Age, years (SD)	23.96 (3.38)	1	23.95 (3.67)	0	23.97 (3.18)	1	0.691^b^
Semester, years (SD)	5.86 (3.74)	3	5.50 (3.68)	1	6.08 (3.76)	2	0.07^b^
*Studies*							
Economics	199 (35.86%)	0	65 (30.52%)	0	134 (39.18%)	0	0.096^c^
Medicine	27 (4.86%)	0	17 (7.98%)	0	10 (2.92%)	0	
Healthcare management	6 (1.08%)	0	3 (1.41%)	0	3 (0.88%)	0	
Educational sciences	62 (11.17%)	0	23 (10.80%)	0	39 (11.40%)	0	
Humanities	104 (18.74%)	0	38 (17.84%)	0	66 (19.30%)	0	
Natural sciences	74 (13.33%)	0	34 (15.96%)	0	40 (11.70%)	0	
Engineering sciences	41 (7.39%)	0	16 (7.51%)	0	25 (7.31%)	0	
Other studies	42 (7.57%)	0	17 (7.98%)	0	25 (7.31%)	0	
*Marital status*							
Unmarried	516 (92.97%)	0	202 (94.84%)	0	314 (91.81%)	0	0.566^c^
Married	18 (3.24%)	0	5 (2.35%)	0	13 (3.80%)	0	
Widowed	0 (0.00%)	0	0 (0.00%)	0	0 (0.00%)	0	
Divorced	2 (0.36%)	0	0 (0.00%)	0	2 (0.58%)	0	
Not specified	19 (3.42%)	0	6 (2.82%)	0	13 (3.80%)	0	

^a^Chi^2^-test

^b^MWU-test

^c^Fisher’s exact test

Table 3 presents details on the participants in the respective experimental conditions with respect to gender, age, and years of study. Additional individual characteristics of the sample are given in Appendix A.4. The comparison of individual and team market treatments with regard to individual characteristics shows no significant differences. Appendix A.5 analyses the first decision made by an individual in the experiment to shed light on the interaction between individual characteristics and preferences regarding quality choices before these choices are affected by the observed quality choices of other participants.

### Majority voting process

Before analysing cooperative behaviour in individual and team markets, we provide descriptive information on the hospitals’ majority voting process in the team market condition. Such teams are formed by three subjects who decide on hospital quality by a majority voting process. If a majority vote is not achieved in the first majority voting round, the process is continued until at least two subjects choose the same quality level. In total, 1,710 majority voting processes (114 hospitals × 15 periods) were conducted. Although there is no external termination rule, 963 (56.32%) of these majority voting processes were completed after the first round and 408 (23.86%) after the second majority voting round. Only eight (0.47%) majority voting processes ended after 10 or more rounds. The longest majority voting process took 32 rounds until a majority vote was reached.

Table 4 shows the average duration of majority voting processes, i.e. the number of voting rounds required to get a majority vote, alongside the 15 periods of the experiment. The results show that average durations tended to decline over the 15 periods, except for the final period. However, the small increase of average duration in period 15 may be induced by an outlier with 32 majority voting rounds in one hospital.^22^ The average duration of majority voting processes per market of period 1–5 does not differ significantly from the duration of period 6–10 (Mann–Whitney U test [MWU],^23^*p* = 0.062) or from the duration of period 11–15 (MWU, *p* = 0.062) at a 5% significance level. Also the average duration per market of period 6–10 does not differ significantly from the duration of period 11–15 (MWU, *p* = 0.242) at a 5% significance level.

**Table 4:** Average duration of majority voting processes in the team market condition

	Periods					
	1	1–5	6–10	11–15	15	Total
*Team*	2.211	1.937	1.902	1.793	1.833	1.877
SD	1.30	0.697	0.982	1.264	3.016	0.787
N	114	114	114	114	114	

Observations are hospitals. The duration is measured by the number of voting rounds required to get a majority vote

### Degree of cooperation

Following Suetens and Potters (2007) and Potters and Suetens (2009), we analyse cooperative behaviour in our individual and team healthcare markets by presenting our results on collusion in terms of the degree of cooperation (see Table 5). In aggregate, both individual and team markets provide quality levels above the non-cooperative Nash equilibrium, which translate into negative average degrees of cooperation: − 0.038 for individual and − 0.109 for team markets (see Table 5). The average degree of cooperation is statistically significantly different from 0 for teams (Wilcoxon signed-rank test [WSR], *p* = 0.000) and individuals (WSR, *p* = 0.013). This finding is in line with Hypothesis 1 and indicates that markets are, to some degree, patient centred (competitive). The difference in the average degrees of cooperation between individuals and teams is not significant (MWU, *p* = 0.174).

**Table 5:** Average degrees of cooperation on market level

	Periods					
	1	1–5	6–10	11–15	15	Total
*Individual*	0.120	− 0.004	− 0.053	− 0.058	− 0.101	− 0.038
SD	0.246	0.218	0.227	0.259	0.252	0.235
N	71	71	71	71	71	
*Team*	0.128	− 0.049	− 0.131	− 0.149	− 0.175	− 0.109
SD	0.131	0.096	0.121	0.113	0.131	0.118
N	38	38	38	38	38	

N equals the number of hospital markets. An individual market consists of three individual hospitals and a team market of three hospitals with three board members each

**Fig. 1:**
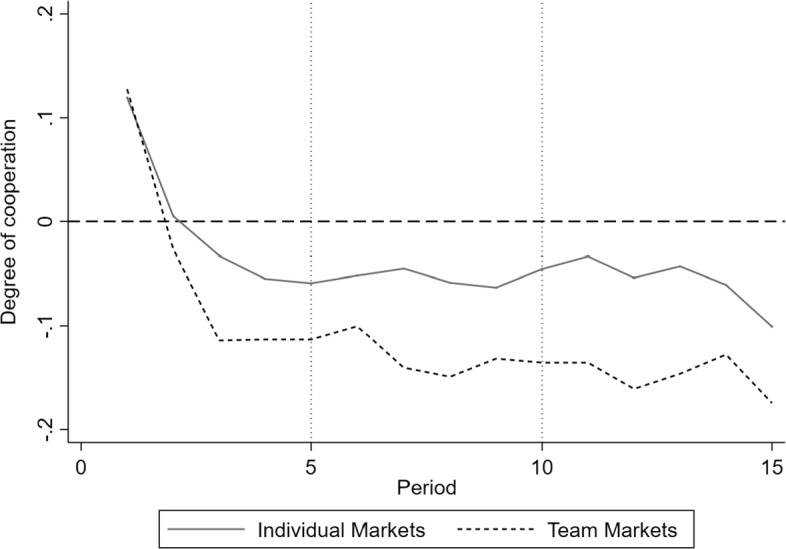
Average degrees of cooperation—individual versus team part 1 by period

Next, we consider the dynamics over the 15 periods. Figure 1 illustrates the average degrees of cooperation for both individual and team markets over the 15 rounds. To account for this variation over time, we calculate means for periods 1–5, 6–10, and 11–15, similar to Huck et al. (2007) (see Table 5). The average degrees of cooperation for both types of markets start in period 1 at similar positive levels around 0.1 and quickly drop to 0 and below over the first five periods. The drop is higher for team conditions, but the difference between conditions is not statistically significant at this stage (MWU, *p* = 0.48).^24^ In the middle stage between periods 6 and 10, both markets establish degrees of cooperation below and significantly different from 0 (individual WSR, *p* = 0.003; team WSR, *p* = 0). The average level of the degrees of cooperation in individual markets is − 0.053 and − 0.131 in team markets, respectively. Hence, team markets show lower degrees of cooperation and potentially more patient centred (competitive) behaviour than individual markets after markets have settled. This difference in cooperation rates between individual and team markets is not significant (MWU, *p* = 0.137).^25^ The final stage between periods 11 and 15 does not differ much from the previous one. While degrees of cooperation differ significantly from 0 (WSR, *p* = 0.003 in individual markets; WSR, *p* = 0 in team markets), the difference of their distribution between individual and teams does not differ significantly (MWU, *p* = 0.237).^26^

Hence, while individual markets show behaviour that is close to non-cooperative profit maximization, hospitals in team markets tend to choose quality levels that are more patient centred (competitive): the average quality level of the final stage is at 10.52 (SD 2.327) for individual markets and 11.34 (SD 1.020) for team markets. Thus, markets in both conditions have average qualities higher than the Nash equilibrium level. The difference between treatments is also not significant (MWU, *p* = 0.242). To some extent, this result is different from the findings in Gillet et al. (2011) for Bertrand quality competitions, which found higher prices with the majority voting rule compared to individual decision making. This should translate into lower quality levels and a higher degree of cooperation in our setting (see Hypothesis 3). The effect in our setting might be offset by semi-altruism.

#### Result 1

(*Corresponding Hypotheses 1 and 3—Degree of Cooperation*) Individual and team markets start at slightly positive degrees of cooperation, but they quickly converge to average levels significantly below 0, indicating patient centred or competitive behaviour. While the average degrees of cooperation (quality levels) are higher (lower) for individual than for team markets, the difference is not significant.

### Coordination behaviour

So far, we have considered the aggregate degree of cooperation. However, the latter does not shed light on market heterogeneity and whether markets coordinate on certain quality levels. To get a better understanding of coordination behaviour within markets, we now consider the difference between hospitals’ quality choices within each market. For this, we consider the absolute difference between the highest and lowest hospital quality of a respective hospital within each market in every period. A small quality spread suggests that hospitals within a market jointly gravitate towards a reference quality level and indicates coordination behaviour. Moreover, the quality spread tends to be larger with hospitals that consider quality as a strategic substitute rather than as a strategic complement. For reference, a profit maximizing deviation from JPM would imply a spread of 4 or 5, and moving from the Nash equilibrium to the patient optimum would imply a spread of 3 (see Table 2).

**Fig. 2:**
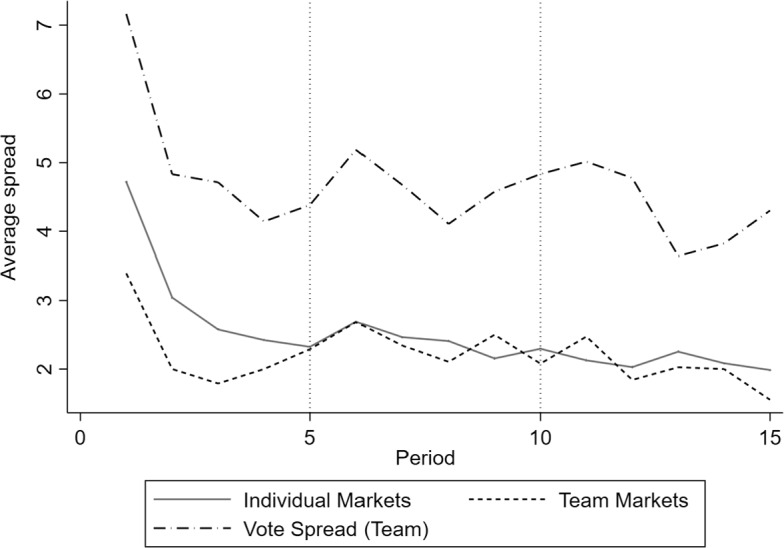
Quality spread in individual and team markets

The average quality spreads in our experimental conditions can be inferred from Table 6 and Fig. 2.^27^ The quality spread is significantly higher for individual decision makers in the first five periods, with 3.02 quality units on average compared to 2.30 in teams (MWU, *p* = 0.003 for periods 1–5). Over time, the spreads converge to similar levels around 2 and are not significantly different anymore (MWU, *p* = 0.889 for periods 5–10; MWU, *p* = 0.957 for periods 10–15). It seems that the team decision process ‘filters out’ extreme decisions in the beginning and creates more stable market qualities overall. The third line in Fig. 2 corresponds to the spread of quality proposals in the majority decision process for the team decisions. As expected, its trend is parallel to the actual team decision. We thereby contribute evidence comparing the coordination behaviour of individuals and teams in competitive markets with semi-altruistic behaviour (see Hypothesis 4).

**Table 6:** Average quality spreads—individual versus team by period intervals on market level

	Periods					
	1	1–5	6–10	11–15	15	Total
*Individual*	4.718	3.017	2.403	2.096	1.986	2.505
SD	2.630	1.288	1.461	1.325	1.938	1.407
N	71	71	71	71	71	
*Team*	3.395	2.295	2.342	1.979	1.553	2.205
SD	2.087	0.865	1.186	0.926	0.978	1.006
N	38	38	38	38	38	

N equals the number of hospital markets. An individual market consists of three individual hospitals and a team market consists of three hospitals with three board members each

**Fig. 3:**
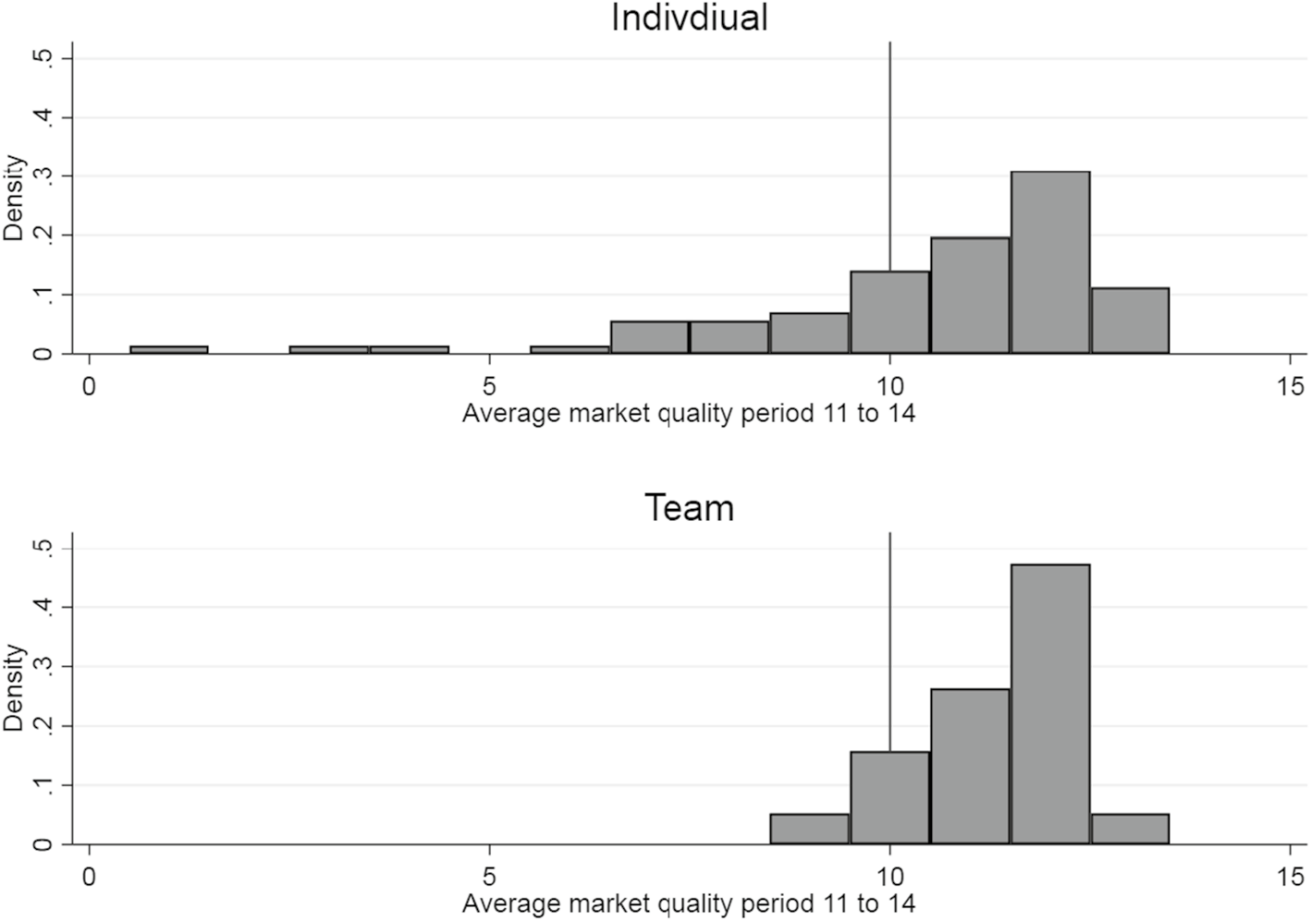
Average market quality between periods 11 and 14

While markets tend to gravitate toward the same quality levels, these quality levels could be different for each market. Figure 3 explores the heterogeneity of resulting average market qualities in periods 11 to 14. Due to end-game effects, period 15 is neglected. The left skewness of the distribution suggests that markets coordinate on levels between the competitive and patient optimal outcome in the final periods.

Using the average standard deviation of market qualities, we find similar results as for quality spreads (see Table 7). The average standard deviation of market qualities is significantly different for team and individual markets (MWU, *p* = 0.001). This result is mostly driven by periods 1–5 (MWU, *p* = 0.000). The average standard deviation of market qualities of periods 6–10 (MWU, *p* = 0.122) and of periods 11–15 (MWU, *p* = 0.208) is not significantly different for team and individual markets.

**Table 7:** Average Standard Deviation—Individual vs. Team Condition by Period Intervals on Market Level

	Periods					
	1	1–5	6–10	11–15	15	Total
*Individual*	2.483	1.603	1.297	1.139	1.064	1.389
SD	1.376	0.657	0.777	0.696	1.018	0.537
N	71	71	71	71	71	
*Team*	1.57	1.068	1.097	0.926	0.755	1.05
SD	0.957	0.402	0.561	0.426	0.467	0.329
N	38	38	38	38	38	

N equals the number of hospital markets. An individual market consists of three individual hospitals and a team market of three hospitals with three board members each

#### Result 2

(*Corresponding Hypothesis 4—Coordination*) Initially, quality levels are more homogeneous in team markets—i.e., the quality spread is significantly higher for individual markets than for team markets. This might be explained by the preceding team decision process, with majority voting filtering out extreme quality choices from the beginning. Towards the final periods, both markets coordinate at levels between the competitive and patient optimal outcome.

### Strategic nature of quality

Having identified some degree of patient centredness (competitiveness), especially for team markets, we now investigate the direction in which hospitals react to their competitors’ average quality choice. For this, we run the fixed effects model separately for individual and team markets:

12 $$ {\Delta }choice_{it} = \beta_{o} + \beta_{1} {\Delta }avg.rivalchoice_{ t - 1} + \nu_{i} + u_{it} . $$

The model investigates how strong and in which direction a hospital $i$ in period $t$ reacts to the average quality change from rivals in the previous period. On the left-hand side, we have the difference in quality choice of hospital $i$ between the previous period $t - 1$ and the current period $t$. The independent variable is the difference in the average quality choice from period $t - 2$ to $t - 1$ by the competing hospitals. We include individual fixed effects. The results are presented in Table 8. Column (1) shows the results for individual markets and column (2) for team markets. From column (1), we can infer that in individual markets the coefficient is positive and statistically significant at the 1 percent level. Hence, as predicted by our model, quality choices are treated as strategic complements. This is in line with Hypothesis 2 and implies that hospitals maximizing their objective function play reciprocally by following the previous market quality levels. From column (2), we can see that the coefficient is still positive but smaller and significant at the 5 percent level. Hence, the effect is weaker in the team conditions, but it is still in line with complementarity.

**Table 8:** Regression results on changes in choices for all, cooperative, and non-cooperative markets

	(1) Individual Markets	(2) Team Markets	(3) Individual Cooperative	(4) Team Cooperative	(5) Individual Non-Cooperative	(6) Team Non-Cooperative
Avg. rivalchange	0.268***	0.095*	0.295***	0.492***	0.248***	0.056
	(0.032)	(0.045)	(0.056)	(0.011)	(0.039)	(0.041)
Constant	0.040***	0.086***	0.022***	-0.006**	0.052***	0.102***
	(0.004)	(0.008)	(0.003)	(0.001)	(0.006)	(0.008)
N	2769	1482	936	156	1833	1326
Adjusted R^2^	0.035	0.004	0.040	0.091	0.031	0.001

Fixed effects model separately for individual and team markets. Results are clustered on market level. Standard errors in parentheses

*p < 0.05; **p < 0.01; ***p < 0.001

Next, we separate markets by their average degree of cooperation—that is, cooperative markets with an average degree of cooperation $\overline{\rho } > 0$ and those with an average degree of cooperation $\overline{\rho } \le 0$. This might give us clearer insight into the relationship between cooperativeness and strategic complementarity. Columns (3) and (4) compare individual and team markets in markets with a positive degree of cooperation. For both, the coefficient is positive and statistically significant at the 1 percent level. Hence, one can infer that both individual and team cooperative markets display strategic complementarity. Columns (5) and (6) consider non-cooperative individual and team markets with an average degree of cooperation $\overline{\rho } \le 0$. Here, we can infer that while the coefficient for non-cooperative individual markets is still highly significant, the one for non-cooperative team markets is not significantly different from 0. Thus, teams in more patient centred (competitive) markets do not seem to be responsive to changes in their rivals’ qualities but tend to set qualities independently. We thereby contribute evidence comparing the strategic nature of quality provision of individuals and teams in competitive markets with semi-altruistic behaviour (see Hypothesis 4).^28^

#### Result 3

(*Corresponding Hypothesis 2—Strategic Nature*) Individual hospital markets treat quality levels as strategic complements and adjust their quality choice in the same direction as their competitors. This also holds for team markets. However, their response magnitude is weaker. This might be driven by non-cooperative or semi-altruistic team markets that are not responsive to changes in their rivals’ qualities but tend to set qualities independently.

## Conclusion

In this paper, we investigated quality competition in healthcare markets where providers are assumed to be altruistic towards patients with a focus on two aspects that are especially relevant from a behavioural perspective in this context: first, how hospitals react to a change in their competitors’ quality choice, and second, how the decision process for quality choices—i.e., individual or team decision made within hospitals—affects quality provision behaviour. We implemented a laboratory experiment based on a Salop model, where hospitals competed for patients via quality provision in a market with regulated prices. The market consisted of three hospitals. We also implemented an experimental condition with a team decision process. Here, hospital boards were made up of three members who had to agree on a quality level via a simple majority rule. Decisions affect real patients outside the laboratory since the generated patient utility is monetized and transferred to a charity that provides medical care to patients without access to formal healthcare.

With respect to the general competition analysis, we do not find much evidence for collusion within competitive healthcare markets. In particular, we show that individual and team markets start at slightly positive degrees of cooperation but then quickly converge towards negative values, indicating patient centred (competitive) behaviour. This is in line with evidence from previous health economic experiments for duopolies of general practitioners by Brosig-Koch et al. (2017b). They show that collusion, while being observed, is less frequent than in related price competition experiments without a health framing.

Comparing individual markets with team markets, we show that while the average degrees of cooperation (quality levels) are descriptively higher (lower) for individual than for team markets, the difference is not statistically significant. To some extent, this result is different from Gillet et al. (2011) who find higher prices for Bertrand quality competitions, which should translate into lower quality levels and a higher degree of cooperation in our setting, with the majority voting rule compared to individual decision making. The effect in our setting might be offset by semi-altruistic behaviour. We also show that initially, quality levels are significantly more homogeneous in team markets than in individual ones. This might be explained by the preceding team decision process with majority voting filtering out extreme quality choices from the beginning. This is in line with evidence from Bornstein and Gneezy (2002) for team markets and price competition without altruism, showing that team markets stabilize much quicker than individual markets. Also, Bornstein et al. (2008) find that team markets tend to behave more stable overall. Towards the final periods, however, we show that both types of markets coordinate at levels between the competitive and patient optimal outcome. Potters and Suetens (2009) find similar endgame effects with negative degrees of cooperation for strategic substitutes and degrees close to the competitive benchmark for complements in the final period in markets without semi-altruistic clients.

We also consider strategic behaviour and find that hospitals led by an individual treat quality as a strategic complement and adjust their quality choice in the same direction as their competitors. This also holds for hospitals led by teams. However, their response magnitude is weaker, driven by non-cooperative or altruistic team markets, which do not seem to be responsive to changes in their rivals’ quality levels but tend to set quality levels independently. Our result is in line with previous evidence by Potters and Suetens (2009) for non-collusive markets in games with strategic substitutes. It reinforces the idea that cooperation is easier when goods are seen as strategic complements, as Holt (1995) and Suetens and Potters (2007) suggest. This finding can also be linked to the theoretical considerations of Brekke et al. (2017) regarding competitive healthcare markets with altruistic providers. They argue that quality is a strategic complement as long as altruism is relatively low compared to the cost substitutability between quality and output, while strong altruism could yield quality as a strategic substitute. However, we do not find such extreme results except for our most altruistic markets—i.e., non-cooperative team markets. Here, quality is no longer treated as a complement but is strategically independent. We thereby also contribute to recent field studies investigating the strategic nature of quality within hospital markets. In line with our results, they find either no response to rivals’ quality in hospital settings or some evidence for complementarity for a few selected quality indicators (Gravelle et al., 2014; Longo et al., 2017). Given our experimental results, this might be driven by the hospitals’ degree of cooperativeness or altruism within the market.

Overall, our results contribute novel experimental evidence regarding competition in healthcare markets. While the previous literature suggests that the higher the degree of observability of hospital quality indicators for patients, the stronger the hospitals’ response to a rival hospital’s quality (see, for example, Gravelle et al., 2014), our findings suggest that hospitals’ strategic quality provision behaviour might also depend on the degree of altruism and the organizational structure—that is, whether the quality decisions of the hospital are made by individuals or by teams. While regulators often foster quality improvement in healthcare with incentives for competition—e.g., by addressing market concentration or public reporting of quality data—the organizational structure of hospitals might be an additional channel to consider.

Future research could analyse in more detail to what extent individual characteristics (e.g. socio-demographics, risk attitudes, and personality traits) might change the overall findings on the strategic nature of quality. Since relevant characteristics can differ for the individual market situation and the majority voting process, this could be helpful in finding out what drives quality decisions in the respective situations.

## Supplementary Information

Below is the link to the electronic supplementary material.

Supplementary file1 (PDF 3350 kb)

## Footnotes

1 Note that Bloom (2015) finds that competition has a positive impact on outcome as well as management quality in the English public hospital sector, yet management quality is measured by a survey tool covering things apart from the decision-making processes, including incentives, monitoring, or target setting.

2 Economists have used laboratory experiments to study competition in markets for quite some time (see Holt, 1995 and Potters & Suetens, 2013 for a more recent example). The majority of these experiments is either set in a quantity or price competition environment. An important distinction between games of quantity and games of price setting is that the former are considered as strategic substitutes and the latter as strategic complements in competition. Generally, for strategic substitutes, the optimal response to an aggressive strategy by the competitor is to become less aggressive – i.e., move into the opposite direction – while for strategic complements the optimal response is to become more aggressive and follow the direction of the move (Tirole, 1988).

3 Contributions by Pauly and Redisch (1973) and Harris (1977) address the issue of stakeholders with different objectives in hospitals, while Phelps (2002) investigates different preferences in the ruling body of hospitals when it comes to quality decisions. For a detailed discussion of team decisions in hospitals, see Barros and Olivella (2011).

4 Brosig-Koch et al. (2017a) consider collusive behaviour, but only for two physicians. Han et al. (2017) investigate the effects on quality of a merger due in hospital markets with quality competition and do not investigate coordination behaviour.

5 The evidence has a tendency, but it is ambiguous to some degree. For example, Bornstein and Gneezy (2002) find that teams converge quicker on the competitive solution, while Raab and Schipper (2009) find no differences between individual and team decisions for three firm Cournot oligopolies.

6 To reduce complexity for participants, they were provided with a discrete set of quality levels to choose from, even if the theoretical framework was based on continuous functions.

7 For a comparison of a monopoly and a competitive market, see Han et al. (2017).

8 The assumption of linear demand is a strong one. Changing the demand function possibly changes the prediction of the model. This is a clear limitation of the model and the corresponding experiment.

9 In our model approach, hospitals cannot endogenously choose locations since their locations are fixed.

10 We assume in the following that disutility from traveling is sufficiently small, i.e., $t<3 p/c$.

11 A quality level of zero is excluded in the design as this would mean non-treatment. Treating a patient should at least lead to the lowest quality level possible.

12 Taler is our experimental currency unit. The exchange rates differ for the two conditions: 1 Taler = 0.07€ in the individual market conditions and 1 Taler = 0.21€ in the team market conditions. We do so in order to make individual earnings comparable, although we increase the number of participants per market from one to three in the team conditions. For more detail, see Section "Experimental procedure".

13 Due to the discrete parametrization for our experiment, the best response is ambiguous.

14 For simplicity, we assume homogeneity in the degree of altruism.

15 While it would be interesting to be able to differentiate between the drivers of strategic behaviour, the primary aim of this paper is to identify the latter and to compare the outcomes of markets with an individual decision process to those with a team decision process (see below).

16 An extension of the experimental model framework to teams is beyond the scope of this paper, which focusses on the experiment.

17 Most surveys indicate that teams are typically closer to the standard game theory predictions than individuals are (see, e.g., Bornstein, 2008; Cooper & Kagel, 2005; Kugler et al., 2012). However, there is also a small number of studies reporting team decisions to be less selfish and rational compared to individual decisions (see, e.g., Kocher & Sutter, 2007; Müller & Tran, 2013).

18 Of the participants, 286 were female and 269 were male. Our sample included 27 medical students and 199 business and economics students.

19 Individual and team conditions had different durations, so sessions were held separately. Also, we only used the observations from the first part for this study.

20 In the comprehension questions, we first checked for whether participants understood how to read the profit and patient benefit tables. For this, they were given an example part of profit and patient benefit tables with different numbers than in the experiment. They had to indicate the profits of their own hospital and the other hospitals, as well as the respective patient benefit levels for a given quality of care. The other questions were true and false questions to check for the understanding of their role as decision maker. For an example, see the translated screenshots of the comprehension questions for the individual condition in Appendix A.2.4.

21 The remaining participants were from other fields of study, including healthcare management (1.08%), educational sciences (11.17%), humanities (18.74%), natural sciences (13.33%).

22 The mean duration of majority voting process in period 15 would be 1.566 (SD 0.990) without the outlier.

23 It is always two-sided unless indicated otherwise.

24 Standard deviations are continuously higher in the individual markets than in the team markets. There seems to be a larger variety of different markets in terms of degree of cooperation when individuals are in charge. We will have a closer look at this variety in Appendix A.6. The lower standard deviation also suggests that the voting process in team decisions aligns the voting behaviour.

25 While we cannot reject an equality in distributions with the MWU, there are still some relevant differences between conditions. The majority of markets in both conditions have negative degrees of cooperation (69 percent of individual and 82 percent of team markets) with almost identical average levels of -0.17 for individuals and -0.18 for teams. However, among markets with positive degrees of cooperation, the average degree of cooperation for individuals is much higher at 0.21 compared to teams with 0.07. In other words, uncooperative markets are very similar between conditions, but cooperative markets are much more cooperative in the individual conditions – but there are not too many. So, if we focused on the means and take a two-sided t-test, we would reject the hypothesis of equality of means with p = 0.049. This will also be addressed in Appendix A.6 when we look at cooperative markets and collusion.

26 Again, taking means into account changes the test results slightly (two-sided t-test, p = 0.042).

27 The relatively high quality and vote spreads in the respective individual and team markets in the first period indicate that we do not have an anchoring effect. In case of anchoring on, e.g. the Nash equilibrium or patient optimal quality levels, we should see much lower spreads.

28 A random effects model on changes in choices, which clusters on market level, yields very similar results.
