# Supplementary material for: Strategic behaviour and decision making in competitive hospital markets: an experimental investigation
Source: Int J Health Econ Manag. 2024 Mar 15;24(3):333–55. doi: 10.1007/s10754-024-09366-3 (PMC11445323; doi:10.1007/s10754-024-09366-3)

Strategic behaviour and decision making in competitive hospital markets: an experimental investigation

08 March 2024

Johann Han, Nadja Kairies-Schwarz, Markus Vomhof

APPENDIX

A.1 Experimental Design

A.1.1 Profit Tables and Patient Utility Tables

Profit table

quality level of the other two hospitals / own quality level	1|1	2|2	3|3	4|4	5|5	6|6	7|7	8|8	9|9	10|10	11|11	12|12	13|13
1	**14.00** / 14.00 / 14.00	**12.83** / 13.89 / 13.89	**11.67** / 13.72 / 13.72	**10.50** / 13.50 / 13.50	**9.33** / 13.22 / 13.22	**8.17** / 12.89 / 12.89	**7.00** / 12.50 / 12.50	**5.83** / 12.06 / 12.06	**4.67** / 11.56 / 11.56	**3.50** / 11.00 / 11.00	**2.33** / 10.39 / 10.39	**1.17** / 9.72 / 9.72	**0.00** / 9.00 / 9.00
2	**14.44** / 13.42 / 13.42	**13.33** / 13.33 / 13.33	**12.22** / 13.19 / 13.19	**11.11** / 13.00 / 13.00	**10.00** / 12.75 / 12.75	**8.89** / 12.44 / 12.44	**7.78** / 12.08 / 12.08	**6.67** / 11.67 / 11.67	**5.56** / 11.19 / 11.19	**4.44** / 10.67 / 10.67	**3.33** / 10.08 / 10.08	**2.22** / 9.44 / 9.44	**1.11** / 8.75 / 8.75
3	**14.78** / 12.83 / 12.83	**13.72** / 12.78 / 12.78	**12.67** / 12.67 / 12.67	**11.61** / 12.50 / 12.50	**10.56** / 12.28 / 12.28	**9.50** / 12.00 / 12.00	**8.44** / 11.67 / 11.67	**7.39** / 11.28 / 11.28	**6.33** / 10.83 / 10.83	**5.28** / 10.33 / 10.33	**4.22** / 9.78 / 9.78	**3.17** / 9.17 / 9.17	**2.11** / 8.50 / 8.50
4	**15.00** / 12.25 / 12.25	**14.00** / 12.22 / 12.22	**13.00** / 12.14 / 12.14	**12.00** / 12.00 / 12.00	**11.00** / 11.81 / 11.81	**10.00** / 11.56 / 11.56	**9.00** / 11.25 / 11.25	**8.00** / 10.89 / 10.89	**7.00** / 10.47 / 10.47	**6.00** / 10.00 / 10.00	**5.00** / 9.47 / 9.47	**4.00** / 8.89 / 8.89	**3.00** / 8.25 / 8.25
5	**15.11** / 11.67 / 11.67	**14.17** / 11.67 / 11.67	**13.22** / 11.61 / 11.61	**12.28** / 11.50 / 11.50	**11.33** / 11.33 / 11.33	**10.39** / 11.11 / 11.11	**9.44** / 10.83 / 10.83	**8.50** / 10.50 / 10.50	**7.56** / 10.11 / 10.11	**6.61** / 9.67 / 9.67	**5.67** / 9.17 / 9.17	**4.72** / 8.61 / 8.61	**3.78** / 8.00 / 8.00
6	**15.11** / 11.08 / 11.08	**14.22** / 11.11 / 11.11	**13.33** / 11.08 / 11.08	**12.44** / 11.00 / 11.00	**11.56** / 10.86 / 10.86	**10.67** / 10.67 / 10.67	**9.78** / 10.42 / 10.42	**8.89** / 10.11 / 10.11	**8.00** / 9.75 / 9.75	**7.11** / 9.33 / 9.33	**6.22** / 8.86 / 8.86	**5.33** / 8.33 / 8.33	**4.44** / 7.75 / 7.75
7	**15.00** / 10.50 / 10.50	**14.17** / 10.56 / 10.56	**13.33** / 10.56 / 10.56	**12.50** / 10.50 / 10.50	**11.67** / 10.39 / 10.39	**10.83** / 10.22 / 10.22	**10.00** / 10.00 / 10.00	**9.17** / 9.72 / 9.72	**8.33** / 9.39 / 9.39	**7.50** / 9.00 / 9.00	**6.67** / 8.56 / 8.56	**5.83** / 8.06 / 8.06	**5.00** / 7.50 / 7.50
8	**14.78** / 9.92 / 9.92	**14.00** / 10.00 / 10.00	**13.22** / 10.03 / 10.03	**12.44** / 10.00 / 10.00	**11.67** / 9.92 / 9.92	**10.89** / 9.78 / 9.78	**10.11** / 9.58 / 9.58	**9.33** / 9.33 / 9.33	**8.56** / 9.03 / 9.03	**7.78** / 8.67 / 8.67	**7.00** / 8.25 / 8.25	**6.22** / 7.78 / 7.78	**5.44** / 7.25 / 7.25
9	**14.44** / 9.33 / 9.33	**13.72** / 9.44 / 9.44	**13.00** / 9.50 / 9.50	**12.28** / 9.50 / 9.50	**11.56** / 9.44 / 9.44	**10.83** / 9.33 / 9.33	**10.11** / 9.17 / 9.17	**9.39** / 8.94 / 8.94	**8.67** / 8.67 / 8.67	**7.94** / 8.33 / 8.33	**7.22** / 7.94 / 7.94	**6.50** / 7.50 / 7.50	**5.78** / 7.00 / 7.00
10	**14.00** / 8.75 / 8.75	**13.33** / 8.89 / 8.89	**12.67** / 8.97 / 8.97	**12.00** / 9.00 / 9.00	**11.33** / 8.97 / 8.97	**10.67** / 8.89 / 8.89	**10.00** / 8.75 / 8.75	**9.33** / 8.56 / 8.56	**8.67** / 8.31 / 8.31	**8.00** / 8.00 / 8.00	**7.33** / 7.64 / 7.64	**6.67** / 7.22 / 7.22	**6.00** / 6.75 / 6.75
11	**13.44** / 8.17 / 8.17	**12.83** / 8.33 / 8.33	**12.22** / 8.44 / 8.44	**11.61** / 8.50 / 8.50	**11.00** / 8.50 / 8.50	**10.39** / 8.44 / 8.44	**9.78** / 8.33 / 8.33	**9.17** / 8.17 / 8.17	**8.56** / 7.94 / 7.94	**7.94** / 7.67 / 7.67	**7.33** / 7.33 / 7.33	**6.72** / 6.94 / 6.94	**6.11** / 6.50 / 6.50
12	**12.78** / 7.58 / 7.58	**12.22** / 7.78 / 7.78	**11.67** / 7.92 / 7.92	**11.11** / 8.00 / 8.00	**10.56** / 8.03 / 8.03	**10.00** / 8.00 / 8.00	**9.44** / 7.92 / 7.92	**8.89** / 7.78 / 7.78	**8.33** / 7.58 / 7.58	**7.78** / 7.33 / 7.33	**7.22** / 7.03 / 7.03	**6.67** / 6.67 / 6.67	**6.11** / 6.25 / 6.25
13	**12.00** / 7.00 / 7.00	**11.50** / 7.22 / 7.22	**11.00** / 7.39 / 7.39	**10.50** / 7.50 / 7.50	**10.00** / 7.56 / 7.56	**9.50** / 7.56 / 7.56	**9.00** / 7.50 / 7.50	**8.50** / 7.39 / 7.39	**8.00** / 7.22 / 7.22	**7.50** / 7.00 / 7.00	**7.00** / 6.72 / 6.72	**6.50** / 6.39 / 6.39	**6.00** / 6.00 / 6.00

Patient utlity table

quality level of the other two hospitals / own quality level	1|1	2|2	3|3	4|4	5|5	6|6	7|7	8|8	9|9	10|10	11|11	12|12	13|13
1	**1.00** / 1.00 / 1.00	**0.99** / 1.34 / 1.34	**0.97** / 1.71 / 1.71	**0.94** / 2.09 / 2.09	**0.89** / 2.50 / 2.50	**0.83** / 2.93 / 2.93	**0.75** / 3.38 / 3.38	**0.66** / 3.84 / 3.84	**0.56** / 4.33 / 4.33	**0.44** / 4.84 / 4.84	**0.31** / 5.38 / 5.38	**0.16** / 5.93 / 5.93	**0.00** / 6.50 / 6.50
2	**1.35** / 1.00 / 1.00	**1.33** / 1.33 / 1.33	**1.30** / 1.69 / 1.69	**1.25** / 2.07 / 2.07	**1.19** / 2.47 / 2.47	**1.11** / 2.89 / 2.89	**1.02** / 3.33 / 3.33	**0.92** / 3.79 / 3.79	**0.80** / 4.27 / 4.27	**0.67** / 4.78 / 4.78	**0.52** / 5.30 / 5.30	**0.36** / 5.85 / 5.85	**0.19** / 6.41 / 6.41
3	**1.75** / 0.99 / 0.99	**1.72** / 1.32 / 1.32	**1.67** / 1.67 / 1.67	**1.60** / 2.04 / 2.04	**1.53** / 2.43 / 2.43	**1.44** / 2.84 / 2.84	**1.33** / 3.28 / 3.28	**1.22** / 3.73 / 3.73	**1.08** / 4.21 / 4.21	**0.94** / 4.70 / 4.70	**0.78** / 5.22 / 5.22	**0.60** / 5.76 / 5.76	**0.42** / 6.32 / 6.32
4	**2.19** / 0.97 / 0.97	**2.14** / 1.29 / 1.29	**2.08** / 1.64 / 1.64	**2.00** / 2.00 / 2.00	**1.91** / 2.39 / 2.39	**1.81** / 2.79 / 2.79	**1.69** / 3.22 / 3.22	**1.56** / 3.67 / 3.67	**1.41** / 4.14 / 4.14	**1.25** / 4.63 / 4.63	**1.08** / 5.14 / 5.14	**0.89** / 5.67 / 5.67	**0.69** / 6.22 / 6.22
5	**2.67** / 0.94 / 0.94	**2.60** / 1.26 / 1.26	**2.53** / 1.60 / 1.60	**2.44** / 1.95 / 1.95	**2.33** / 2.33 / 2.33	**2.22** / 2.73 / 2.73	**2.08** / 3.15 / 3.15	**1.94** / 3.59 / 3.59	**1.78** / 4.06 / 4.06	**1.60** / 4.54 / 4.54	**1.42** / 5.04 / 5.04	**1.22** / 5.57 / 5.57	**1.00** / 6.11 / 6.11
6	**3.19** / 0.91 / 0.91	**3.11** / 1.22 / 1.22	**3.02** / 1.55 / 1.55	**2.92** / 1.90 / 1.90	**2.80** / 2.27 / 2.27	**2.67** / 2.67 / 2.67	**2.52** / 3.08 / 3.08	**2.36** / 3.51 / 3.51	**2.19** / 3.97 / 3.97	**2.00** / 4.44 / 4.44	**1.80** / 4.94 / 4.94	**1.58** / 5.46 / 5.46	**1.35** / 6.00 / 6.00
7	**3.75** / 0.88 / 0.88	**3.66** / 1.18 / 1.18	**3.56** / 1.50 / 1.50	**3.44** / 1.84 / 1.84	**3.31** / 2.21 / 2.21	**3.16** / 2.59 / 2.59	**3.00** / 3.00 / 3.00	**2.83** / 3.43 / 3.43	**2.64** / 3.88 / 3.88	**2.44** / 4.34 / 4.34	**2.22** / 4.83 / 4.83	**1.99** / 5.34 / 5.34	**1.75** / 5.88 / 5.88
8	**4.35** / 0.83 / 0.83	**4.25** / 1.13 / 1.13	**4.13** / 1.44 / 1.44	**4.00** / 1.78 / 1.78	**3.85** / 2.14 / 2.14	**3.69** / 2.51 / 2.51	**3.52** / 2.91 / 2.91	**3.33** / 3.33 / 3.33	**3.13** / 3.77 / 3.77	**2.92** / 4.24 / 4.24	**2.69** / 4.72 / 4.72	**2.44** / 5.22 / 5.22	**2.19** / 5.75 / 5.75
9	**5.00** / 0.78 / 0.78	**4.88** / 1.07 / 1.07	**4.75** / 1.38 / 1.38	**4.60** / 1.70 / 1.70	**4.44** / 2.06 / 2.06	**4.27** / 2.43 / 2.43	**4.08** / 2.82 / 2.82	**3.88** / 3.23 / 3.23	**3.67** / 3.67 / 3.67	**3.44** / 4.12 / 4.12	**3.19** / 4.60 / 4.60	**2.94** / 5.09 / 5.09	**2.67** / 5.61 / 5.61
10	**5.69** / 0.72 / 0.72	**5.56** / 1.00 / 1.00	**5.41** / 1.30 / 1.30	**5.25** / 1.63 / 1.63	**5.08** / 1.97 / 1.97	**4.89** / 2.33 / 2.33	**4.69** / 2.72 / 2.72	**4.47** / 3.13 / 3.13	**4.24** / 3.55 / 3.55	**4.00** / 4.00 / 4.00	**3.74** / 4.47 / 4.47	**3.47** / 4.96 / 4.96	**3.19** / 5.47 / 5.47
11	**6.42** / 0.65 / 0.65	**6.27** / 0.93 / 0.93	**6.11** / 1.22 / 1.22	**5.94** / 1.54 / 1.54	**5.75** / 1.88 / 1.88	**5.55** / 2.23 / 2.23	**5.33** / 2.61 / 2.61	**5.10** / 3.01 / 3.01	**4.86** / 3.43 / 3.43	**4.60** / 3.87 / 3.87	**4.33** / 4.33 / 4.33	**4.05** / 4.82 / 4.82	**3.75** / 5.32 / 5.32
12	**7.19** / 0.58 / 0.58	**7.03** / 0.85 / 0.85	**6.85** / 1.14 / 1.14	**6.67** / 1.44 / 1.44	**6.47** / 1.77 / 1.77	**6.25** / 2.13 / 2.13	**6.02** / 2.50 / 2.50	**5.78** / 2.89 / 2.89	**5.52** / 3.30 / 3.30	**5.25** / 3.74 / 3.74	**4.97** / 4.19 / 4.19	**4.67** / 4.67 / 4.67	**4.35** / 5.16 / 5.16
13	**8.00** / 0.50 / 0.50	**7.83** / 0.76 / 0.76	**7.64** / 1.04 / 1.04	**7.44** / 1.34 / 1.34	**7.22** / 1.67 / 1.67	**6.99** / 2.01 / 2.01	**6.75** / 2.38 / 2.38	**6.49** / 2.76 / 2.76	**6.22** / 3.17 / 3.17	**5.94** / 3.59 / 3.59	**5.64** / 4.04 / 4.04	**5.33** / 4.51 / 4.51	**5.00** / 5.00 / 5.00

A.1.2 Explanation of Sample Sizes

At the time the experiments were conducted, it was not common to do a power analysis. In Han et al. (2017) we therefore based the sample size calculations on the related literature on market experiments and their common number of participants per treatment. Potters and Suetens (2009), e.g. had 13-14 independent observations (pairs) per treatment, and Raab and Schipper (2009) had 9-6 matching groups per treatment.

Although such a rather low number of observations was common during that time when analyzing markets which consist of groups, we aimed at a higher number of observations per treatment that was relatable to experiments with individual decision makers during that time, i.e. about 24 observations per treatment.

In Han et al. (2017), we employed 4 treatments with individual markets (n=3 per market) which were identical in the first part, yet differed in the second part of the experiment. For each of these 4 treatments we had 23 to 24 observations yielding a total number of 71 market observations for individual market treatments. The control treatment with team markets taken from Han et al. (2017) had 13 observations (n=9 per market) and left little room to investigate strategic behavior. We therefore, decided to run additional sessions to increase the number of team market observations. Although it would have been ideal to have a comparable number of observations, i.e. about 70, our budget as well as subject pool were limited. We therefore ran as many sessions as both allowed yielding an additional 25 market observations, i.e. 225 additional participants. Overall, 555 individuals participated in this experiment.

A.2 Instructions, Screenshots, and Ex-post Questionnaire

A.2.1 Instructions Individual and Team Conditions (Part 1)

General Instructions for all of the Participants (translated into English from German)

The original experiment consisted of two parts, where part 2 differed between conditions. We only use part 1 for this study. Part 1 was identical for all experimental conditions. Parts of the instructions that were exclusive to the *team condition*, are written in *italics.* Parts that were exclusive to the **individual condition** are marked [**bold and in squared brackets**.] Everything else is identical.

<ins>Preliminary remarks</ins>

You are participating in a study of choice behavior for the purpose of experimental economic research. During the experiment you and the other participants are asked to make decisions. In doing so, you can earn money. The resulting amount depends on your decisions and the decisions of the other participants. After finishing the experiment, your total earnings will be converted into Euro and paid out to you in cash. For this experiment all amounts are designated in Taler, the laboratory currency, where 1 Taler translates to *0.21* **[0.07]** Euro.

The experiment will last approximately 150 minutes and consists of two parts. Prior to each of the two parts, you will receive detailed instructions. Note, that neither your decisions made in the first part nor the decisions made in the second part will have an influence on the respective other part. Moreover, there are neither right nor wrong answers in any of the two parts.

Part 1

Please read the following instructions carefully. In case you have any questions along the course of the experiment, please feel free to call attention for yourself by raising your hand. We will come to you to answer the open questions.

<ins>Situation: A hospital market</ins>

In this experiment, *you are part of a hospital board with three members and jointly* **[you are in charge of a hospital and]** decide on the quality level of your hospital for the treatment of patients. Apart from you, two other hospitals, *which are also led by hospital boards with three members in charge of quality decisions,* operate in the same market. The three hospitals are

equidistantly located. Patients are uniformly allocated between the hospitals. The figure illustrates the hospital market.

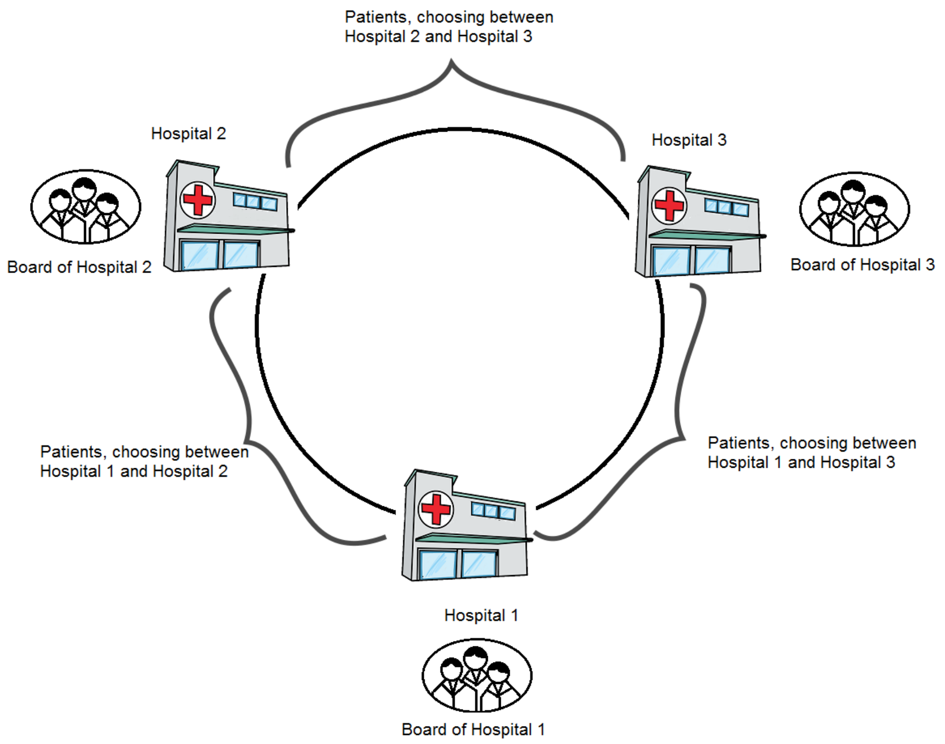

Figure in Team Conditions

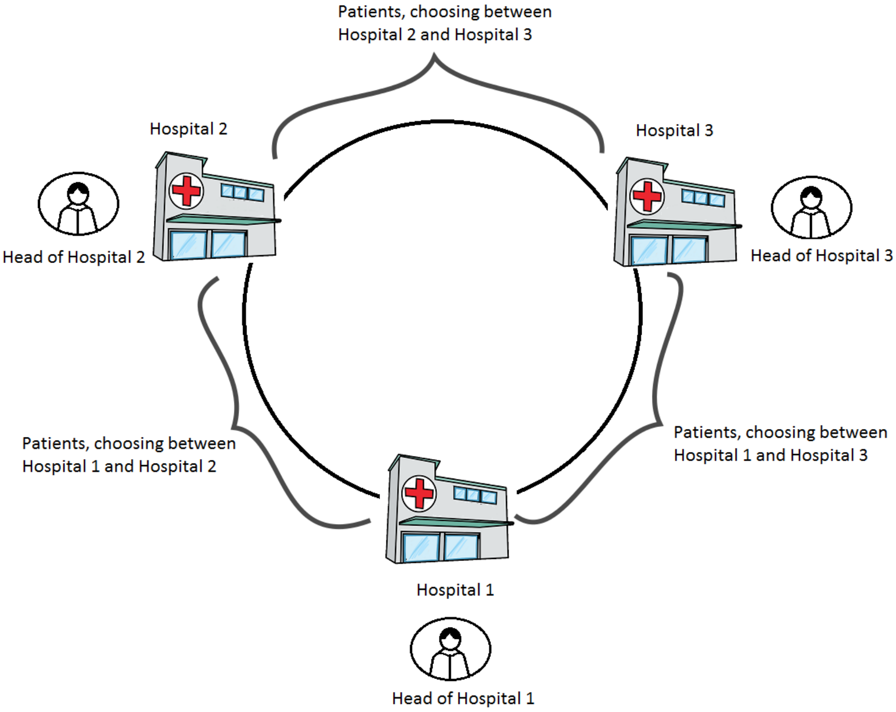

Figure in Individual Conditions

The profit of your hospital depends on the number of treated patients. Due to the uniform distribution, the number of patients in the catchment area of each hospital is equal. Patients prefer short distances and high quality levels. Thus, patients will choose their hospitals by weighing quality levels against the distance of the respective hospital.

The location of your hospital is fixed, hence the quality level is your only means to make yourself attractive to patients. Accordingly, the number of treated patients will depend on the quality level of your hospital relative to the quality levels of the other two hospitals.

As catchment areas of the hospitals overlap, competition for patients can occur. Given equal distances, a patient will choose the hospital with the higher quality level. The preference for short distances also implies that a patient located between two hospitals will always choose one of these two.

In the beginning of the experiment, you will be randomly matched to a hospital market with *eight other* **[two other]** participants for the course of the experiment. *Two of them will be on the board of the hospital with you. The other six participants will be in the boards of the other two hospitals. Each participant decides on the hospital's quality level in the role of a board*

member. [**Each participant will be in charge of a hospital and decide on the hospital's quality level.]**

<u>Description of decision rounds</u>

There are 15 decision rounds in part 1. Each round is payment relevant and your quality decisions will affect your own profit and an amount of money that is paid to real patients outside the lab.

In each round, you have to choose the quality level of your hospital *with the other two board members*. The other *two hospital boards* **[heads]** simultaneously make their own quality choices for their respective hospital. You can choose between 13 quality levels (1,2,3, … , 11,12,13), where 1 is the lowest and 13 is the highest quality level. You can only pick integers. A higher quality level is associated with higher costs. Since patients prefer higher quality levels, you can influence the number of patients treated at your hospital by the quality choice. Thus, the market shares of the three hospitals depend on their relative quality levels. Hospitals receive a fixed payment for each treated patient.

Patient benefit is composed of the quality level of the hospital where they are treated minus the cost of traveling to the hospital. Hence, patient benefit increases with higher quality levels and decreases with higher costs of travel. In this experiment it will be displayed as a monetary value and also called "patient utility". The sum of patient utility will be paid out to real patients after the experiment ends.

For an illustration of this context and as assistance for your decision making a profit- and patient utility-table as well as a profit and benefit calculator are provided to you.

<u>Profit- and patient utility table</u>

The profit table and patient utility table were handed out with the instructions. It serves to give you an overview of the context.

From the profit table you can learn how profits are distributed among the hospitals given the choice of the other two hospitals. Costs from higher quality and changes in market shares are already included. The bold values with white background color in each row display your hospital's profit for each quality level. The profit depends on your quality level relative to the ones of the other two hospitals in the market. The grey shaded columns display the profits of the other two hospital given that they choose uniform quality levels.

The profit is shared evenly among the hospital board members. In other words, your own payment equals one third of the values in the table.

The patient utility table provides you with information about contributions to patient utility given various choices of quality level. The structure and way of reading is equivalent to the profit table. For different combination you can learn about the contributions of a hospital to patient utility given uniform quality choices of the other two hospitals. The patient utility is shown as a monetary value and can be interpreted as a payment to the patient.
Correspondingly, the table gives you an overview how decisions about quality levels affect the profits of hospitals and patient utility.
Please note that the tables only provide you with information for uniform quality choices of the other two hospitals. Cases where the other two hospitals choose different quality levels can be simulated with the profit- and patient utility calculator.

<u>Profit- and patient utility calculator</u>
A calculator will be displayed on the left hand side of your screen in every decision round. You can use this calculator to calculate profits and patient utility for every possible constellation, i.e. situations which are included in the tables as well as those not included. You can use the calculator by typing in your quality choice and hypothetical choices of the *other two hospital boards* **[participants]** and clicking "Calculate". The profits and respective contributions to patient utility of your hospital and the other two hospitals will be displayed afterwards. Like the tables, the calculator simply serves to assist you in your decision-making.

<u>Decision about quality level</u>
When you have decided on the quality level, type in your choice on the right hand side of the screen and propose it to your other two board members by clicking „Propose". They will also submit their quality proposals. After all three proposals have been submitted, they will be shown to all board members. In case of two (or more) equal proposals, this proposal is chosen and determined to be the quality level of the hospital for this round. In case of three different quality proposals, the voting procedure is repeated until a majority for a quality proposal is obtained.
[When you have decided on the quality level, type in your choice in the field „Quality level" on the right hand side of the screen and click „Select". The heads of the other two hospitals will also choose their quality levels.]

<u>Information</u>

Patients` hospital choice is simulated based on the decisions of the three hospitals after every round. Then, you receive information about the other two hospital's quality levels and the resulting profits for every hospital. *The profit is split evenly between the board members. Your individual payment equals one third of the hospital profit and will be displayed as well.* Additionally, you will be informed about your hospital's contribution as well as the other two hospitals' contributions to patient utility. From the history table you can derive the hospitals' individual decisions, corresponding profits and contributions to patient utilities.

Payment

At the end of the experiment the profits of each round are summed up and converted in Euro. Each participant is paid *his individual share of hospital* [**out the profit of his hospital]** profit in cash.

For this part of the experiment, no patients are physically present in the laboratory. The patient utility generated by your hospital's quality choices in each round goes to real patients: The sum all rounds will be transferred to domestic projects of Ärzte der Welt e.V. (Doctors of the World), 80807 Munich. In these projects individuals in Germany without health insurance or access to the regular healthcare system receive basic medical treatment. Ärzte der Welt e.v. holds the DZI Spendensiegel (DZI seal of approval) which certifies transparent, goal oriented and economic use of donations.

The transfer of money to the Ärzte der Welt e.V. will be carried out by the experimenter and one control person after the experiment. The control person completes a money transfer form by filling in the total patient benefit (in Euro) resulting from the realized contributions to patient utilities over all decision rounds. The payment of this amount is then handled by the finance department of the University of Duisburg-Essen, which will transfer the payment from the designated budget for this experiment to the Ärzte der Welt e.V. The form is then sealed in a stamped envelope and deposited in the nearest mailbox by the control person and and the experimenter.

After the part 2 of the experiment is completed, one participant is randomly chosen to be the control person. The control person receives an additional compensation of 5 Euro for this task. The control person certifies that the process has been completed as described here by signing a statement that can be looked at by all participants at the office of the health economics research center CINCH. Moreover, upon request you may also take a look at the donation receipt from Ärzte der Welt e.V.

Comprehension questions

Prior to the decision rounds we kindly ask you to answer a few comprehension questions. These comprehension questions are intended to help you familiarize yourself with the decision situations. In case you have any questions, please raise your hand. Part 1 of the experiment will begin once all participants have answered the comprehension questions correctly.

Original Instructions in German – Individual Condition

Willkommen zum Experiment!

Vorbemerkungen

Sie nehmen an einer Untersuchung von Entscheidungsverhalten im Rahmen der experimentellen Wirtschaftsforschung teil. Während des Experiments werden Sie und die anderen Teilnehmer gebeten, Entscheidungen zu treffen. Dabei können Sie Geld verdienen. Wie viel Geld das sein wird, hängt von Ihren Entscheidungen und den Entscheidungen der anderen Teilnehmer ab. Am Ende des Experiments wird Ihr gesamter Verdienst in Euro umgerechnet und Ihnen bar ausgezahlt. In diesem Experiment werden alle Beträge in Taler, der Laborwährung, ausgewiesen, wobei gilt, dass:

1 Taler = 0,07 Euro.

Das Experiment dauert etwa 150 Minuten und besteht aus zwei Teilen. Vor jedem der beiden Teile des Experiments erhalten Sie detaillierte Instruktionen. Beachten Sie, dass weder Ihre Entscheidungen im ersten Teil des Experiments noch Ihre Entscheidungen im zweiten Teil des Experiments einen Einfluss auf den jeweils anderen Teil des Experiments haben werden. Außerdem gibt es in beiden Teilen weder richtige noch falsche Antworten.

Teil 1

Bitte lesen Sie die nachfolgenden Instruktionen sorgfältig durch. Sollten Sie während des Experiments Fragen haben, können Sie sich zu jedem Zeitpunkt durch Handzeichen melden. Wir werden dann zu Ihnen kommen.

<u>Situation: Ein Krankenhaus-Markt</u>

In diesem Experiment entscheiden Sie als Leiter eines Krankenhauses über dessen Qualitätsstandard bei der Behandlung von Patienten. Neben Ihnen sind noch zwei weitere Krankenhäuser mit jeweils einem Leiter im Markt, die ebenfalls jeweils Entscheidungen über die Qualitätsstandards treffen. Die drei Krankenhäuser sind räumlich in gleichen Entfernungen voneinander gelegen. Die Patienten sind gleichmäßig zwischen den Krankenhäusern angesiedelt. Die Grafik illustriert den Krankenhausmarkt.

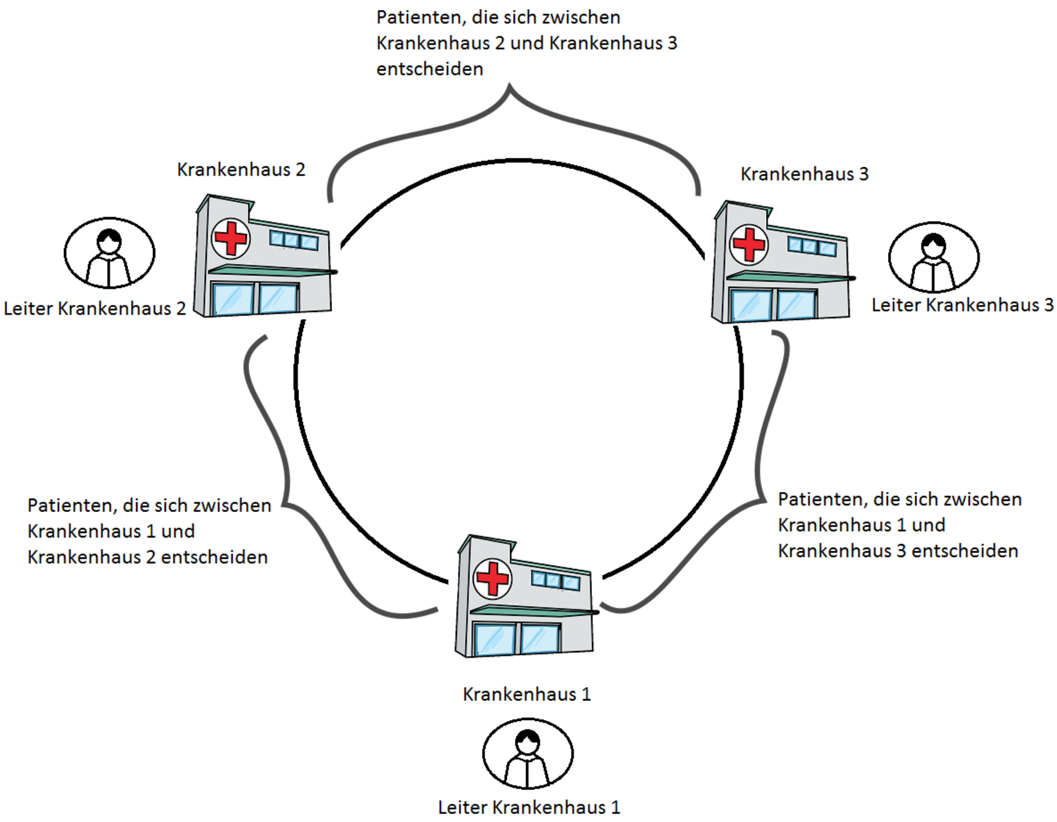

Der Gewinn Ihres Krankenhauses bestimmt sich durch die Anzahl der behandelten Patienten. Durch die gleichmäßige Verteilung der Patienten hat jedes Krankenhaus gleichviele Patienten in seinem Einzugsbereich. Patienten bevorzugen einen kurzen Weg und einen hohen

Qualitätsstandard. Die Entscheidung, zu welchem Krankenhaus ein Patient geht, trifft er, indem er zwischen dem jeweiligen Qualitätsstandard und der Distanz zum jeweiligen Krankenhaus abwägt.

Der Standort Ihres Krankenhauses ist unveränderbar, d.h. Sie können sich nur über den Qualitätsstandard Ihres Krankenhauses für Patienten attraktiver machen. Die tatsächliche Patientenzahl hängt also von dem Qualitätsstandard Ihres Krankenhauses relativ zu dem Qualitätsstandard der zwei anderen Krankenhäuser ab.

Da sich die Einzugsgebiete der Krankenhäuser überschneiden, kann dies dazu führen, dass Sie um einige Patienten konkurrieren. Bei gleicher Distanz wird sich ein Patient für das Krankenhaus mit dem höheren Qualitätsstandard entscheiden. Die Präferenz für kurze Wege impliziert zudem, dass ein Patient, der zwischen zwei Krankenhäusern wohnt, sich immer zwischen einem dieser beiden Krankenhäuser entscheiden wird.

Zu Beginn des Experiments werden Sie zunächst für die Dauer des Experiments zufällig mit zwei anderen Teilnehmern zu einem Krankenhausmarkt zugeordnet. Jeder Teilnehmer trifft in der Rolle eines Krankenhausleiters Entscheidungen über den Qualitätsstandard seines Krankenhauses.

Beschreibung der Entscheidungsrunden

Teil 1 besteht aus 15 Entscheidungsrunden. Jede Runde ist auszahlungsrelevant und Ihre Qualitätsentscheidungen beeinflussen die Höhe Ihres Verdienstes sowie die Höhe einer Auszahlung, die an reale Patienten außerhalb des Labors erfolgt.

Sie müssen in jeder Entscheidungsrunde den Qualitätsstandard Ihres Krankenhauses festlegen. Die anderen beiden Leiter entscheiden ebenfalls jeweils simultan für ihr Krankenhaus. Bei der Wahl des Qualitätsstandards stehen Ihnen 13 Stufen zur Auswahl (1,2,3,…11,12,13), wobei 1 den niedrigsten und 13 den höchsten Qualitätsstandard darstellt. Sie können nur ganze Zahlen wählen. Ein höherer Qualitätsstandard ist mit höheren Kosten verbunden. Da Patienten jedoch einen höheren Qualitätsstandard bevorzugen, können Sie durch die Wahl Ihres Qualitätsstandards auch Ihren Anteil, d.h. die Anzahl an Patienten beeinflussen, die zur Behandlung in Ihr Krankenhaus kommen. Die jeweiligen Anteile der drei Krankenhäuser auf dem Markt hängen also von deren Qualitätsstandards relativ zueinander ab. Krankenhäuser erhalten pro behandeltem Patienten eine fixe Zahlung.

Das Wohl des Patienten setzt sich aus dem Nutzen durch den Qualitätsstandard des behandelnden Krankenhauses abzüglich der Kosten für die Reise zum Krankenhaus zusammen. Das Wohl der Patienten steigt somit mit dem Qualitätsstandard und sinkt in den Kosten für die

Reise. Wie der Gewinn der Krankenhäuser wird es in diesem Experiment monetär erfasst und im Folgenden „Patientennutzen“ genannt. Dieser Patientennutzen wird nach dem Experiment an reale Patienten ausgezahlt.

Als Veranschaulichung dieser Zusammenhänge und Entscheidungshilfe für die Wahl Ihres Qualitätsstandards stehen Ihnen eine Gewinn- und Patientennutzentabelle sowie ein Gewinn- und Patientennutzenrechner zur Verfügung.

Gewinn- und Patientennutzentabelle

Die Gewinntabelle und die Patientennutzentabelle wurden Ihnen zusammen mit diesen Instruktionen ausgeteilt. Sie dienen Ihnen dazu einen Überblick über die Zusammenhänge zu bekommen.

Aus der Gewinntabelle können Sie ablesen, wie sich die Gewinne für jeden Qualitätsstandard gegeben der Entscheidung der anderen beiden Krankenhausleiter auf die einzelnen Krankenhäuser verteilen. Dabei sind die Kosten durch einen höheren Qualitätsstandard und entsprechende Veränderungen der jeweiligen Anteile an Patienten bereits berücksichtigt. Die fett gedruckten und weiß hinterlegten Werte in jeder Zeile der Tabelle zeigen Ihnen den Gewinn Ihres Krankenhauses für eine gewählte Qualitätsstufe an. Der Gewinn hängt von der Höhe Ihres Qualitätsstandards relativ zu denen der beiden anderen Krankenhäuser im Markt ab. Den grau hinterlegten Spalten sind die Gewinne der beiden anderen Krankenhäuser zugeordnet, falls beide den gleichen Qualitätsstandard wählen.

Die Patientennutzentabelle gibt Ihnen entsprechend die Beiträge zum Patientennutzen für unterschiedliche Entscheidungen über den Qualitätsstandard an. Sie ist vom Aufbau und der Lesart äquivalent zur Gewinntabelle. Für unterschiedliche Kombinationen können Sie hier ablesen, wieviel die einzelnen Krankenhäuser gegeben ihrer Qualitätsstandards zum gesamten Patientennutzen beitragen, falls die beiden anderen Krankenhäuser den gleichen Qualitätsstandard wählen. Dieser Nutzen ist wie der Krankenhausgewinn monetär erfasst und kann also als Auszahlung für die Patienten interpretiert werden.

Entsprechend bietet Ihnen die Tabelle eine Übersicht darüber, wie sich Entscheidungen über den Qualitätsstandard auf die Gewinne der Krankenhäuser und den Patientennutzen auswirken. Bitte beachten Sie, dass die Tabellen Ihnen nur Informationen über Kombinationen liefern, in denen die anderen Krankenhausleiter beide die gleichen Qualitätsstandards wählen. Die Situation mit unterschiedlichen Entscheidungen der anderen beiden Krankenhäuser können Sie im Gewinn- und Patientennutzenrechner simulieren.

Gewinn- und Patientennutzenrechner

Auf Ihrem Bildschirm ist zudem in jeder Entscheidungsrunde auf der linken Hälfte ein Rechner zu sehen. Mit diesem Rechner können Sie die Gewinne und die Beiträge zum Patientennutzen für alle Situationen berechnen, d.h. sowohl Situationen, die in den Tabellen erfasst sind, als auch diejenigen, die nicht erfasst sind. Sie verwenden den Rechner, indem Sie die eigene Wahl und hypothetische Entscheidungen der anderen Beiden in die jeweiligen Felder eingeben und auf „Berechnen" klicken. Daraufhin werden Ihnen die Gewinne für Ihr Krankenhaus und der beiden anderen Krankenhäuser, sowie die korrespondierenden Beiträge zum Patientennutzen angezeigt. Der Rechner dient Ihnen wie die Tabellen lediglich zur Entscheidungsfindung.

Entscheidung über den Qualitätsstandard

Sobald Sie sich für eine Höhe des Qualitätsstandards entschieden haben, geben Sie diese in das Fenster „Qualitätsstandard" auf der rechten Bildschirmhälfte ein und drücken „Wählen". Die Leiter der anderen Krankenhäuser entscheiden ebenfalls über ihren Qualitätsstandard.

Information

Basierend auf den Entscheidungen der drei Krankenhäuser wird nach jeder Runde die Krankenhauswahl der Patienten simuliert. Sie erhalten danach Informationen über die Qualitätsstandards der anderen beiden Krankenhäuser und den daraus resultierenden Gewinn für Ihr Krankenhaus und den der anderen. Zudem erhalten Sie die Information über den Beitrag Ihres Krankenhauses zum Patientennutzen sowie den der anderen beiden Krankenhäuser. Aus dem Verlauf können Sie die Entscheidungen der einzelnen Krankenhäuser mit den korrespondierenden Gewinnen und Beiträgen zum Patientennutzen über alle gespielten Entscheidungsrunden ablesen.

Auszahlung

Nach Abschluss des Experiments werden die Gewinne der Krankenhäuser über alle Runden aufsummiert und in Euro umgerechnet. Jedem Teilnehmer wird der Gewinn seines Krankenhauses in bar ausgezahlt.

In diesem Teil des Experiments sind keine Teilnehmer in der Rolle des Patienten im Labor anwesend. Der Nutzen, den Patienten aus der Wahl des Qualitätsstandards Ihres Krankenhauses in den jeweiligen Entscheidungsrunden erhalten, wird realen Patienten zu Gute kommen: Der Betrag, der sich über die Runden ergibt, wird zweckgebunden den Inlandsprojekten von Ärzte der Welt e.V., 80807 München, zur Verfügung gestellt. In diesen Projekten wird Menschen in Deutschland ohne Krankenversicherung und Zugang zum Gesundheitssystem eine

medizinische Basisversorgung ermöglicht. Ärzte der Welt e.V. trägt das DZI-Spendensiegel, welches eine transparente, zweckgerichtete und wirtschaftliche Verwendung der Spenden bescheinigt.

Die Übermittlung des Betrages an Ärzte der Welt e.V. wird nach dem Experiment vom Experimentator gemeinsam mit einer Kontrollperson durchgeführt. Die Kontrollperson trägt den Betrag in Euro, der sich aus den realisierten Beiträgen zum Patientennutzen aller Entscheidungsrunden ergibt, in ein Formular zur Zahlungsverfügung an Ärzte der Welt ein. Die Zahlung des Betrags aus den für dieses Experiment vorgesehenen Mitteln wird dann von der Finanzverwaltung der Universität Duisburg-Essen an Ärzte der Welt veranlasst. Das Formular wird in einen an die Finanzverwaltung der Universität Duisburg-Essen adressierten und frankierten Umschlag gesteckt. Dieser Umschlag wird gemeinsam von der Kontrollperson und dem Experimentator in den nächstgelegenen Briefkasten geworfen.

Nach Teil 2 des Experiments wird einem der Teilnehmer zufällig die Rolle der Kontrollperson zugelost. Die Kontrollperson erhält neben der Auszahlung aus dem Experiment eine zusätzliche Aufwandsentschädigung in Höhe von 5 Euro. Die Kontrollperson bestätigt durch ihre Unterschrift in einem für alle Teilnehmer im Sekretariat des gesundheitsökonomischen Forschungszentrum CINCH (Schützenbahn 70, Raum SM 209) einzusehenden Formular, dass sie die an sie gestellten, hier beschriebenen Aufgaben korrekt erfüllt hat. Darüber hinaus können Sie auf Anfrage im Sekretariat eine Bestätigung von Ärzte der Welt über den Zahlungseingang einsehen.

Verständnisfragen

Vor den Entscheidungsrunden möchten wir Sie bitten, einige Verständnisfragen zu beantworten. Diese Verständnisfragen sollen es Ihnen erleichtern, sich mit der Entscheidungssituation vertraut zu machen. Falls Sie dazu Fragen haben, melden Sie sich bitte mit Handzeichen. Nach korrekter Beantwortung der Verständnisfragen durch alle Teilnehmer startet Teil 1 des Experiments.

Original Instructions in German – Team Condition

Willkommen zum Experiment!

Vorbemerkungen

Sie nehmen an einer Untersuchung von Entscheidungsverhalten im Rahmen der experimentellen Wirtschaftsforschung teil. Während des Experiments werden Sie und die anderen Teilnehmer gebeten, Entscheidungen zu treffen. Dabei können Sie Geld verdienen. Wie viel Geld das sein wird, hängt von Ihren Entscheidungen und den Entscheidungen der anderen Teilnehmer ab. Am Ende des Experiments wird Ihr gesamter Verdienst in Euro umgerechnet und Ihnen bar ausgezahlt. In diesem Experiment werden alle Beträge in Taler, der Laborwährung, ausgewiesen, wobei gilt, dass:

1 Taler = 0,21 Euro.

Das Experiment dauert etwa 150 Minuten und besteht aus zwei Teilen. Vor jedem der beiden Teile des Experiments erhalten Sie detaillierte Instruktionen. Beachten Sie, dass weder Ihre Entscheidungen im ersten Teil des Experiments noch Ihre Entscheidungen im zweiten Teil des Experiments einen Einfluss auf den jeweils anderen Teil des Experiments haben werden. Außerdem gibt es in beiden Teilen weder richtige noch falsche Antworten.

Teil 1

Bitte lesen Sie die nachfolgenden Instruktionen sorgfältig durch. Sollten Sie während des Experiments Fragen haben, können Sie sich zu jedem Zeitpunkt durch Handzeichen melden. Wir werden dann zu Ihnen kommen.

Situation: Ein Krankenhaus-Markt

In diesem Experiment entscheiden Sie als Teil einer dreiköpfigen Krankenhausleitung über den Qualitätsstandard des Krankenhauses bei der Behandlung von Patienten. Neben Ihnen sind noch zwei weitere Krankenhäuser mit jeweils einer dreiköpfigen Krankenhausleitung, die Entscheidungen trifft, im Markt. Die drei Krankenhäuser sind räumlich in gleichen Entfernungen voneinander gelegen. Die Patienten sind gleichmäßig zwischen den Krankenhäusern angesiedelt. Die Grafik illustriert den Krankenhausmarkt.

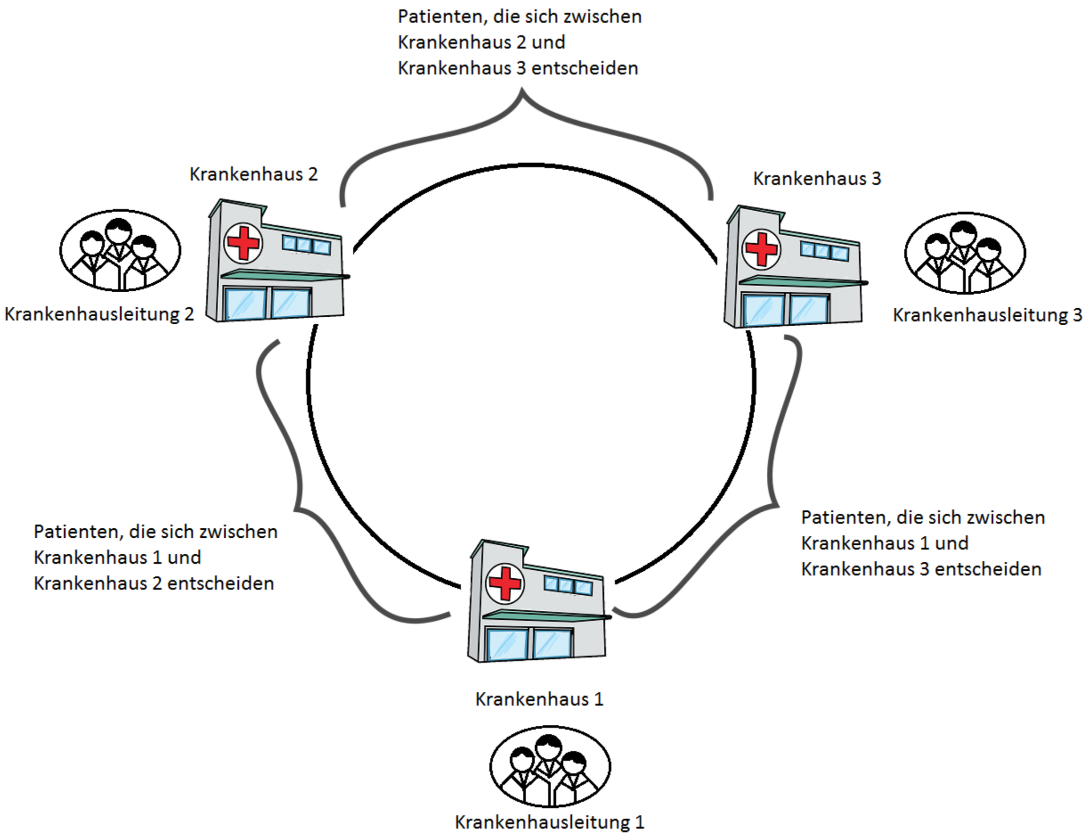

Der Gewinn Ihres Krankenhauses bestimmt sich durch die Anzahl der behandelten Patienten. Durch die gleichmäßige Verteilung der Patienten hat jedes Krankenhaus gleichviele Patienten in seinem Einzugsbereich. Patienten bevorzugen einen kurzen Weg und einen hohen

Qualitätsstandard. Die Entscheidung, zu welchem Krankenhaus ein Patient geht, trifft er, indem er zwischen dem jeweiligen Qualitätsstandard und der Distanz zum jeweiligen Krankenhaus abwägt.

Der Standort Ihres Krankenhauses ist unveränderbar, d.h. Sie können sich nur über den Qualitätsstandard Ihres Krankenhauses für Patienten attraktiver machen. Die tatsächliche Patientenzahl hängt also von dem Qualitätsstandard Ihres Krankenhauses relativ zu dem Qualitätsstandard der zwei anderen Krankenhäuser ab.

Da sich die Einzugsgebiete der Krankenhäuser überschneiden, kann dies dazu führen, dass Sie um einige Patienten konkurrieren. Bei gleicher Distanz wird sich ein Patient für das Krankenhaus mit dem höheren Qualitätsstandard entscheiden. Die Präferenz für kurze Wege impliziert zudem, dass ein Patient, der zwischen zwei Krankenhäusern wohnt, sich immer zwischen einem dieser beiden Krankenhäuser entscheiden wird.

Zu Beginn des Experiments werden Sie zunächst für die Dauer des Experiments zufällig mit acht anderen Teilnehmern zu einem Krankenhausmarkt zugeordnet, von denen zwei zusammen mit Ihnen die Leitung eines der Krankenhäuser übernehmen. Die anderen sechs Teilnehmer bilden entsprechend die Krankenhausleitungen der anderen beiden Krankenhäuser. Jeder Teilnehmer trifft in der Rolle eines Mitglieds der Krankenhausleitung Entscheidungen über den Qualitätsstandard seines Krankenhauses.

Beschreibung der Entscheidungsrunden

Teil 1 besteht aus 15 Entscheidungsrunden. Jede Runde ist auszahlungsrelevant und Ihre Qualitätsentscheidungen beeinflussen die Höhe Ihres Verdienstes sowie die Höhe einer Auszahlung, die an reale Patienten außerhalb des Labors erfolgt.

Sie müssen sich in jeder Entscheidungsrunde mit den anderen beiden Mitgliedern der Krankenhausleitung über den Qualitätsstandard Ihres Krankenhauses einigen. Die anderen beiden Krankenhausleitungen entscheiden ebenfalls jeweils simultan für Ihr Krankenhaus. Bei der Wahl des Qualitätsstandards stehen Ihnen 13 Stufen zur Auswahl (1,2,3,…11,12,13), wobei 1 den niedrigsten und 13 den höchsten Qualitätsstandard darstellt. Sie können nur ganze Zahlen wählen. Ein höherer Qualitätsstandard ist mit höheren Kosten verbunden. Da Patienten jedoch einen höheren Qualitätsstandard bevorzugen, können Sie durch die Wahl Ihres Qualitätsstandards auch Ihren Anteil, d.h. die Anzahl an Patienten beeinflussen, die zur Behandlung in Ihr Krankenhaus kommen. Die jeweiligen Anteile der drei Krankenhäuser auf dem Markt hängen also von deren Qualitätsstandards relativ zueinander ab. Krankenhäuser erhalten pro behandeltem Patienten eine fixe Zahlung.

Das Wohl des Patienten setzt sich aus dem Nutzen durch den Qualitätsstandard des behandelnden Krankenhauses abzüglich der Kosten für die Reise zum Krankenhaus zusammen. Das Wohl der Patienten steigt somit mit dem Qualitätsstandard und sinkt in den Kosten für die Reise. Wie der Gewinn der Krankenhäuser wird es in diesem Experiment monetär erfasst und im Folgenden „Patientennutzen" genannt. Dieser Patientennutzen wird nach dem Experiment an reale Patienten ausgezahlt.

Als Veranschaulichung dieser Zusammenhänge und Entscheidungshilfe für die Wahl Ihres Qualitätsstandards stehen Ihnen eine Gewinn- und Patientennutzentabelle sowie ein Gewinn- und Patientennutzenrechner zur Verfügung.

Gewinn- und Patientennutzentabelle

Die Gewinntabelle und die Patientennutzentabelle wurden Ihnen zusammen mit diesen Instruktionen ausgeteilt. Sie dienen Ihnen dazu einen Überblick über die Zusammenhänge zu bekommen.

Aus der Gewinntabelle können Sie ablesen, wie sich die Gewinne für jeden Qualitätsstandard gegeben der Entscheidung der anderen beiden Krankenhausleitungen auf die einzelnen Krankenhäuser verteilen. Dabei sind die Kosten durch einen höheren Qualitätsstandard und entsprechende Veränderungen der jeweiligen Anteile an Patienten bereits berücksichtigt. Die fett gedruckten und weiß hinterlegten Werte in jeder Zeile der Tabelle zeigen Ihnen den Gewinn Ihres Krankenhauses für eine gewählte Qualitätsstufe an. Der Gewinn hängt von der Höhe Ihres Qualitätsstandards relativ zu denen der beiden anderen Krankenhäuser im Markt ab. Den grau hinterlegten Spalten sind die Gewinne der beiden anderen Krankenhäuser zugeordnet, falls beide den gleichen Qualitätsstandard wählen.

Der Gewinn des Krankenhauses wird zu gleichen Anteilen unter allen Mitgliedern der Krankenhausleitung aufgeteilt. Das heißt, Ihre persönliche Auszahlung entspricht einem Drittel der aufgeführten Werte.

Die Patientennutzentabelle gibt Ihnen entsprechend die Beiträge zum Patientennutzen für unterschiedliche Entscheidungen über den Qualitätsstandard an. Sie ist vom Aufbau und der Lesart äquivalent zur Gewinntabelle. Für unterschiedliche Kombinationen können Sie hier ablesen, wieviel die einzelnen Krankenhäuser gegeben ihrer Qualitätsstandards zum gesamten Patientennutzen beitragen, falls die beiden anderen Krankenhäuser den gleichen Qualitätsstandard wählen. Dieser Nutzen ist wie der Krankenhausgewinn monetär erfasst und kann also als Auszahlung für die Patienten interpretiert werden.

Entsprechend bietet Ihnen die Tabelle eine Übersicht darüber, wie sich Entscheidungen über den Qualitätsstandard auf die Gewinne der Krankenhäuser und den Patientennutzen auswirken. Bitte beachten Sie, dass die Tabellen Ihnen nur Informationen über Kombinationen liefern, in denen die anderen Krankenhausleitungen beide die gleichen Qualitätsstandards wählen. Die Situation mit unterschiedlichen Entscheidungen der anderen beiden Krankenhäuser können Sie im Gewinn- und Patientennutzenrechner simulieren.

Gewinn- und Patientennutzenrechner

Auf Ihrem Bildschirm ist zudem in jeder Entscheidungsrunde auf der linken Hälfte ein Rechner zu sehen. Mit diesem Rechner können Sie die Gewinne und die Beiträge zum Patientennutzen für alle Situationen berechnen, d.h. sowohl Situationen, die in den Tabellen erfasst sind, als auch diejenigen, die nicht erfasst sind. Sie verwenden den Rechner, indem Sie die eigene Wahl und hypothetische Entscheidungen der anderen beiden Krankenhausleitungen in die jeweiligen Felder eingeben und auf „Berechnen" klicken. Daraufhin werden Ihnen die Gewinne für Ihr Krankenhaus und der beiden anderen Krankenhäuser, sowie die korrespondierenden Beiträge zum Patientennutzen angezeigt. Der Rechner dient Ihnen wie die Tabellen lediglich zur Entscheidungsfindung.

Abstimmung über den Qualitätsstandard

Sobald Sie sich für eine Höhe des Qualitätsstandards entschieden haben, geben Sie diese in das Fenster auf der rechten Bildschirmhälfte ein und schlagen diese durch klicken auf „Vorschlagen" den zwei anderen Mitgliedern Ihrer Krankenhausleitung vor. Diese reichen ebenfalls ihren Qualitätsvorschlag ein. Nachdem alle drei Qualitätsvorschläge eingegangen sind, werden diese allen Mitgliedern der Krankenhausleitung angezeigt. Sollten zwei (oder mehr) Vorschläge übereinstimmen, wird dieser Vorschlag gewählt und als Entscheidung über den Qualitätsstandard Ihres Krankenhauses für diese Runde festgelegt. Wenn drei unterschiedliche Qualitätsvorschläge gemacht werden, wiederholt sich die Abstimmungs-Prozedur bis es für einen Qualitätsvorschlag eine Mehrheit gibt.

Information

Basierend auf den Entscheidungen der drei Krankenhäuser wird nach jeder Runde die Krankenhauswahl der Patienten simuliert. Sie erhalten danach Informationen über die Qualitätsstandards der anderen beiden Krankenhäuser und den daraus resultierenden Gewinn für Ihr Krankenhaus und den der anderen. Der Gewinn wird zwischen den Mitgliedern Ihrer Krankenhausleitung geteilt. Ihr individueller Gewinn entspricht einem Drittel des

Krankenhausgewinns und wird Ihnen ebenfalls angezeigt. Zudem erhalten Sie die Information über den Beitrag Ihres Krankenhauses zum Patientennutzen sowie den der anderen beiden Krankenhäuser. Aus dem Verlauf können Sie die Entscheidungen der einzelnen Krankenhäuser mit den korrespondierenden Gewinnen und Beiträgen zum Patientennutzen über alle gespielten Entscheidungsrunden ablesen.

Auszahlung

Nach Abschluss des Experiments werden die Gewinne der Krankenhäuser über alle Runden aufsummiert und in Euro umgerechnet. Jedem Teilnehmer wird der individuelle Anteil am Gewinn seines Krankenhauses in bar ausgezahlt.

In diesem Teil des Experiments sind keine Teilnehmer in der Rolle des Patienten im Labor anwesend. Der Nutzen, den Patienten aus der Wahl des Qualitätsstandards Ihres Krankenhauses in den jeweiligen Entscheidungsrunden erhalten, wird realen Patienten zu Gute kommen: Der Betrag, der sich über die Runden ergibt, wird zweckgebunden den Inlandsprojekten von Ärzte der Welt e.V., 80807 München, zur Verfügung gestellt. In diesen Projekten wird Menschen in Deutschland ohne Krankenversicherung und Zugang zum Gesundheitssystem eine medizinische Basisversorgung ermöglicht. Ärzte der Welt e.V. trägt das DZI-Spendensiegel, welches eine transparente, zweckgerichtete und wirtschaftliche Verwendung der Spenden bescheinigt.

Die Übermittlung des Betrages an Ärzte der Welt e.V. wird nach dem Experiment vom Experimentator gemeinsam mit einer Kontrollperson durchgeführt. Die Kontrollperson trägt den Betrag in Euro, der sich aus den realisierten Beiträgen zum Patientennutzen aller Entscheidungsrunden ergibt, in ein Formular zur Zahlungsverfügung an Ärzte der Welt ein. Die Zahlung des Betrags aus den für dieses Experiment vorgesehenen Mitteln wird dann von der Finanzverwaltung der Universität Duisburg-Essen an Ärzte der Welt veranlasst. Das Formular wird in einen an die Finanzverwaltung der Universität Duisburg-Essen adressierten und frankierten Umschlag gesteckt. Dieser Umschlag wird gemeinsam von der Kontrollperson und dem Experimentator in den nächstgelegenen Briefkasten geworfen.

Nach Teil 2 des Experiments wird einem der Teilnehmer zufällig die Rolle der Kontrollperson zugelost. Die Kontrollperson erhält neben der Auszahlung aus dem Experiment eine zusätzliche Aufwandsentschädigung in Höhe von 5 Euro. Die Kontrollperson bestätigt durch ihre Unterschrift in einem für alle Teilnehmer im Sekretariat des gesundheitsökonomischen Forschungszentrum CINCH (Schützenbahn 70, Raum SM 209) einzusehenden Formular, dass

sie die an sie gestellten, hier beschriebenen Aufgaben korrekt erfüllt hat. Darüber hinaus können Sie auf Anfrage im Sekretariat eine Bestätigung von Ärzte der Welt über den Zahlungseingang einsehen.

Verständnisfragen

Vor den Entscheidungsrunden möchten wir Sie bitten, einige Verständnisfragen zu beantworten. Diese Verständnisfragen sollen es Ihnen erleichtern, sich mit der Entscheidungssituation vertraut zu machen. Falls Sie dazu Fragen haben, melden Sie sich bitte mit Handzeichen. Nach korrekter Beantwortung der Verständnisfragen durch alle Teilnehmer startet Teil 1 des Experiments.

A.2.2 Screenshots of the Decision Situation for Individual and Team Conditions (Part 1)

Individual Markets

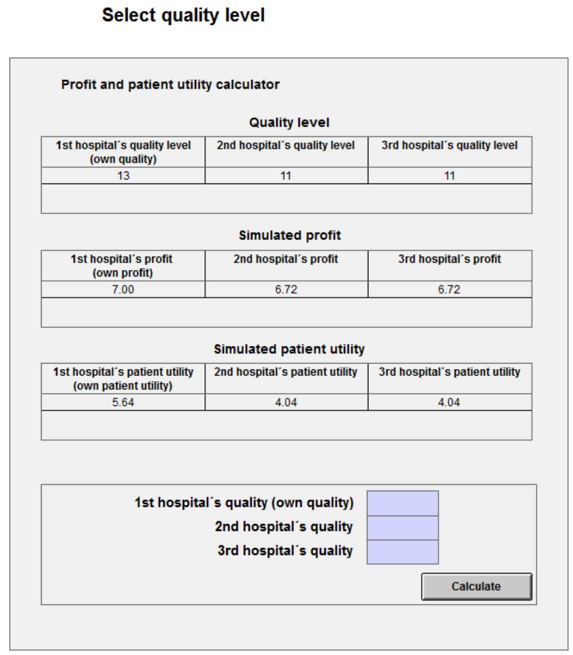

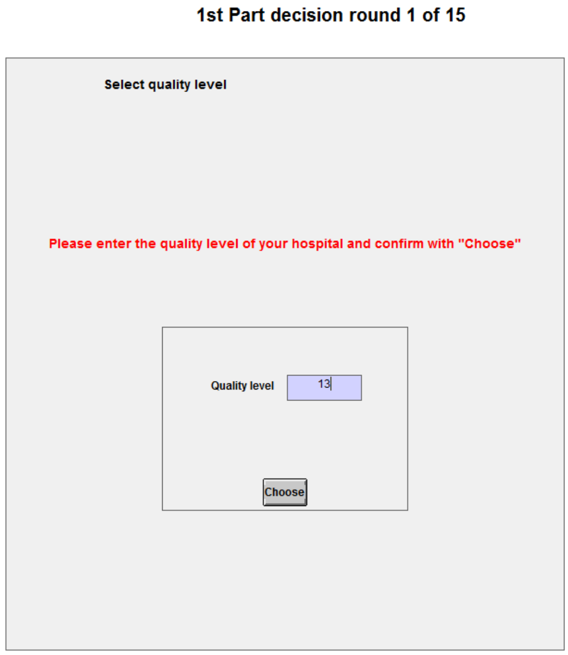

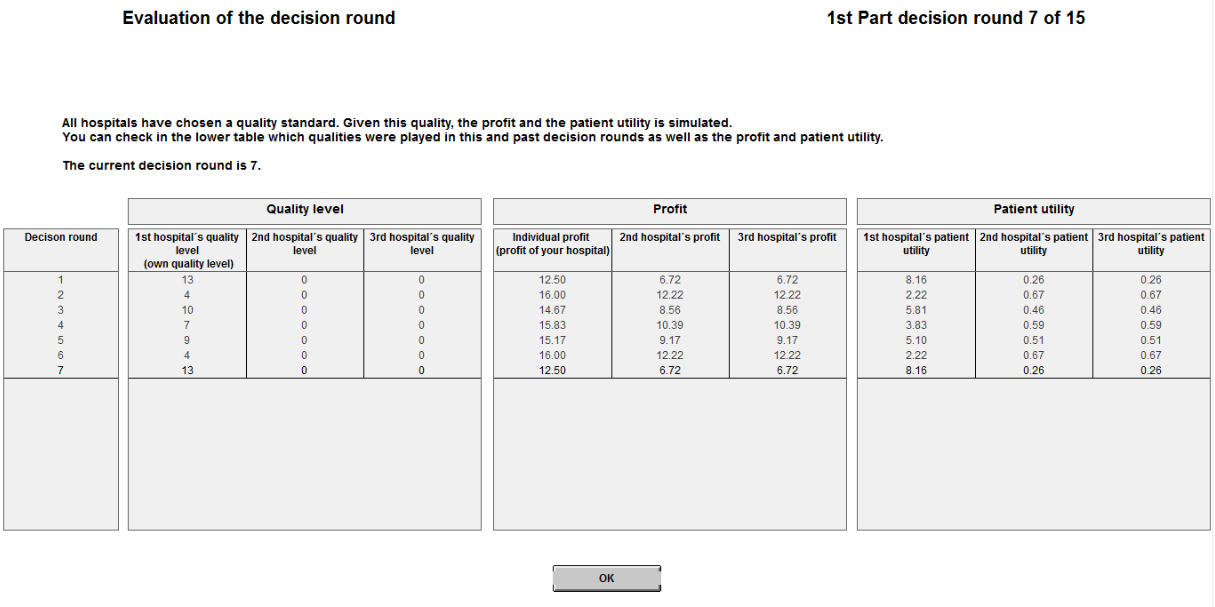

Evaluation of the decision round

1st Part decision round 7 of 15

All hospitals have chosen a quality standard. Given this quality, the profit and the patient utility is simulated.
You can check in the lower table which qualities were played in this and past decision rounds as well as the profit and patient utility.

The current decision round is 7.

	Quality level			Profit			Patient utility		
Decison round	1st hospital´s quality level (own quality level)	2nd hospital´s quality level	3rd hospital´s quality level	Individual profit (profit of your hospital)	2nd hospital´s profit	3rd hospital´s profit	1st hospital´s patient utility	2nd hospital´s patient utility	3rd hospital´s patient utility
1	13	0	0	12.50	6.72	6.72	8.16	0.26	0.26
2	4	0	0	16.00	12.22	12.22	2.22	0.67	0.67
3	10	0	0	14.67	8.56	8.56	5.81	0.46	0.46
4	7	0	0	15.83	10.39	10.39	3.83	0.59	0.59
5	9	0	0	15.17	9.17	9.17	5.10	0.51	0.51
6	4	0	0	16.00	12.22	12.22	2.22	0.67	0.67
7	13	0	0	12.50	6.72	6.72	8.16	0.26	0.26

OK

Team Markets

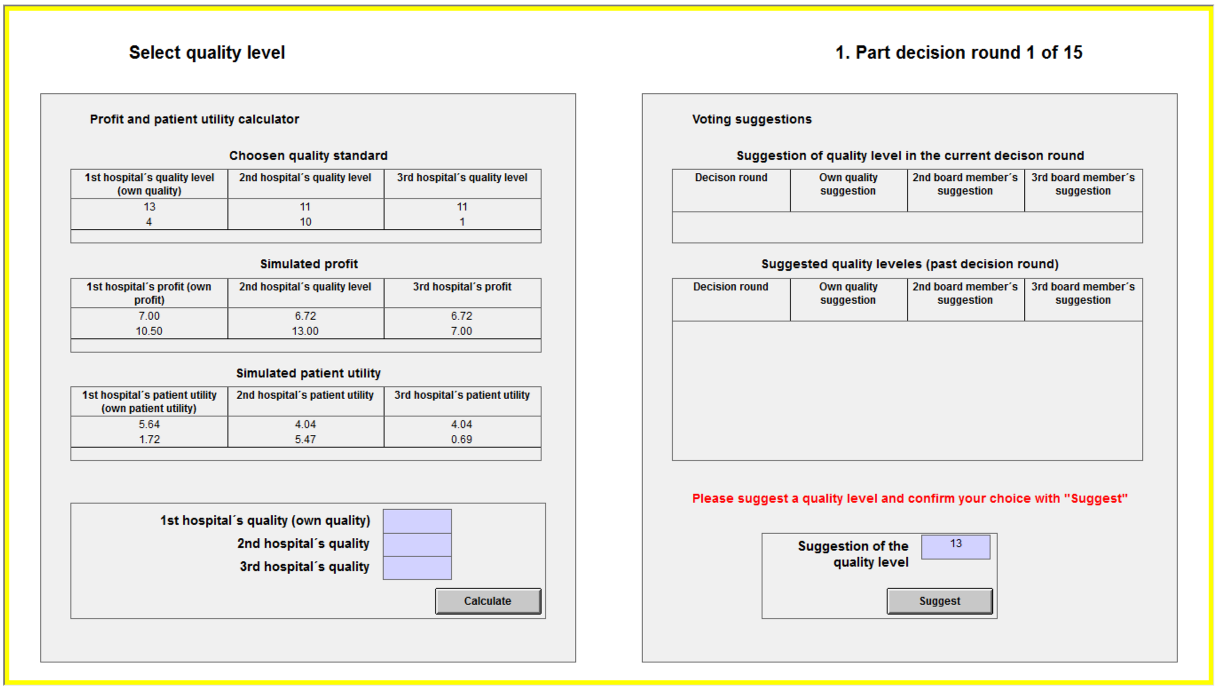

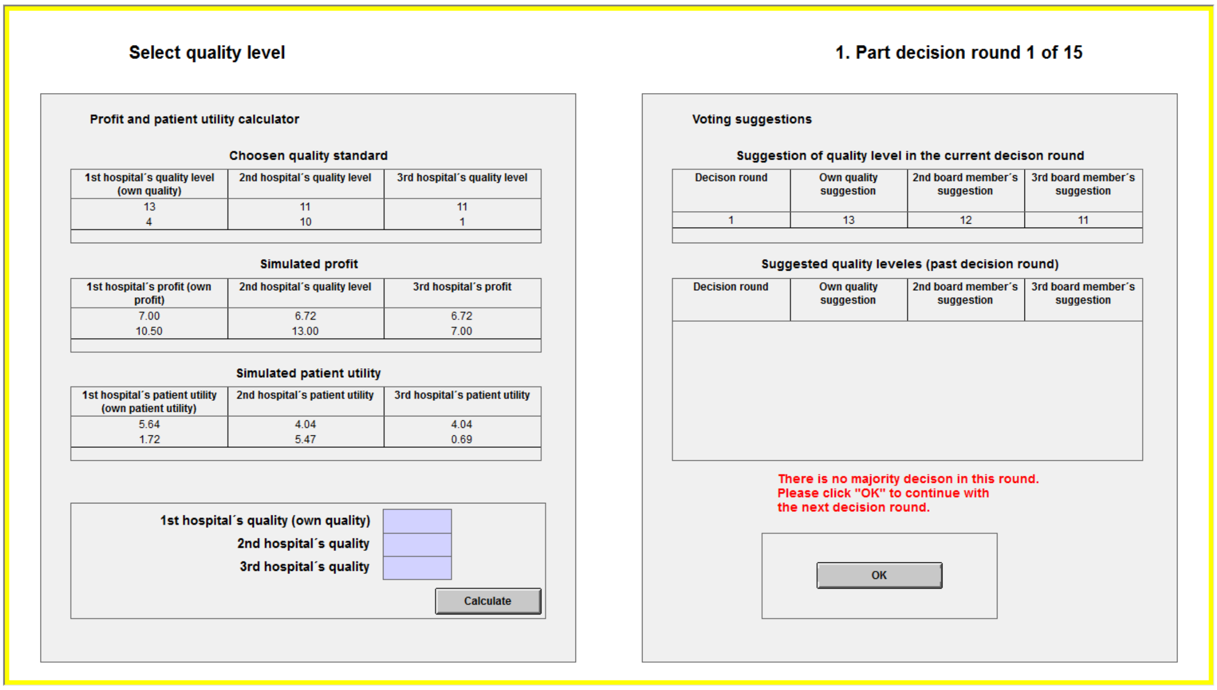

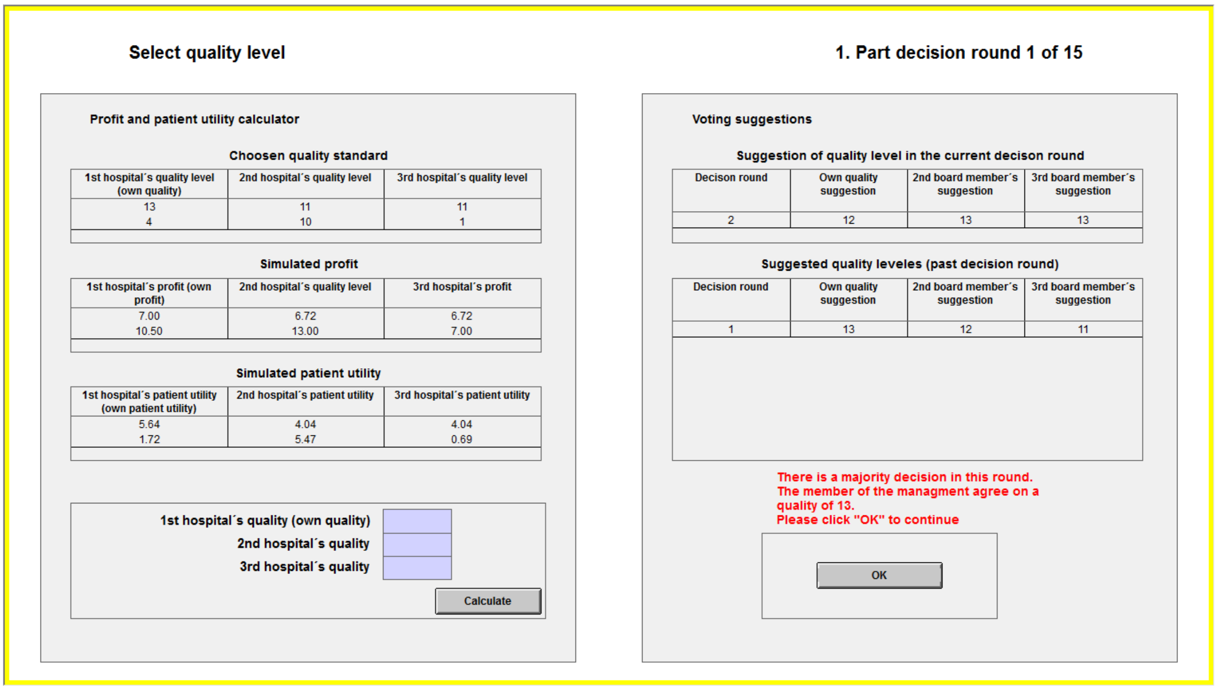
Select quality level
1. Part decision round 1 of 15
Profit and patient utility calculator
Choosen quality standard
1st hospital´s quality level (own quality) | 2nd hospital´s quality level | 3rd hospital´s quality level
13 | 11 | 11
4 | 10 | 1
Simulated profit
1st hospital´s profit (own profit) | 2nd hospital´s quality level | 3rd hospital´s profit
7.00 | 6.72 | 6.72
10.50 | 13.00 | 7.00
Simulated patient utility
1st hospital´s patient utility (own patient utility) | 2nd hospital´s patient utility | 3rd hospital´s patient utility
5.64 | 4.04 | 4.04
1.72 | 5.47 | 0.69
1st hospital´s quality (own quality)
2nd hospital´s quality
3rd hospital´s quality
Calculate
Voting suggestions
Suggestion of quality level in the current decison round
Decison round | Own quality suggestion | 2nd board member´s suggestion | 3rd board member´s suggestion
2 | 12 | 13 | 13
Suggested quality leveles (past decision round)
Decision round | Own quality suggestion | 2nd board member´s suggestion | 3rd board member´s suggestion
1 | 13 | 12 | 11
There is a majority decision in this round.
The member of the managment agree on a quality of 13.
Please click "OK" to continue
OK

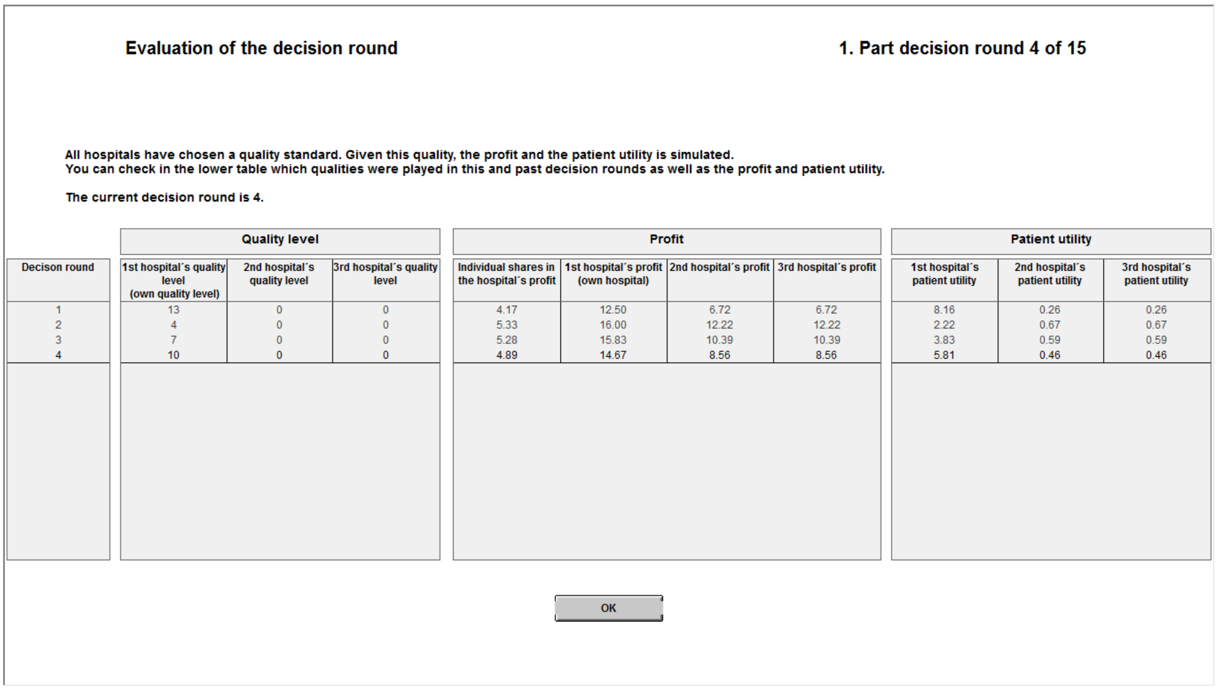
Evaluation of the decision round
1. Part decision round 4 of 15
All hospitals have chosen a quality standard. Given this quality, the profit and the patient utility is simulated.
You can check in the lower table which qualities were played in this and past decision rounds as well as the profit and patient utility.
The current decision round is 4.
Quality level
Profit
Patient utility
Decison round | 1st hospital´s quality level (own quality level) | 2nd hospital´s quality level | 3rd hospital´s quality level | Individual shares in the hospital´s profit | 1st hospital´s profit (own hospital) | 2nd hospital´s profit | 3rd hospital´s profit | 1st hospital´s patient utility | 2nd hospital´s patient utility | 3rd hospital´s patient utility
1 | 13 | 0 | 0 | 4.17 | 12.50 | 6.72 | 6.72 | 8.16 | 0.26 | 0.26
2 | 4 | 0 | 0 | 5.33 | 16.00 | 12.22 | 12.22 | 2.22 | 0.67 | 0.67
3 | 7 | 0 | 0 | 5.28 | 15.83 | 10.39 | 10.39 | 3.83 | 0.59 | 0.59
4 | 10 | 0 | 0 | 4.89 | 14.67 | 8.56 | 8.56 | 5.81 | 0.46 | 0.46
OK

A.2.3 Ex-post Experimental Questionnaire (translated from German)

**Gender**	❑	Male
	❑	Female
**Age**	________	
**Civil status**	❑	unmarried
	❑	married
	❑	widowed
	❑	divorced
	❑	no specification
**Field of Study**	❑	Economics
	❑	Medicine
	❑	Educational Sciences
	❑	Humanities
	❑	Natural Sciences
	❑	Engineering sciences
	❑	Other studies
**In which semester are you studying**	________	

How do you rate yourself personally: Are you generally a person who is impatient or who is always very patient?

Very Patient ❑ ❑ ❑ ❑ ❑ ❑ ❑ ❑ ❑ ❑ ❑ Very Impatient

How do you rate yourself personally: Are you generally a risk-taker or do you try to avoid risks?

Not at all willing to take risks ❑ ❑ ❑ ❑ ❑ ❑ ❑ ❑ ❑ ❑ ❑ Very willing to take risks

You can also behave differently in different areas. How would you rate your risk tolerance in relation to the following areas?

What about investments?

Not at all willing to take risks ❑ ❑ ❑ ❑ ❑ ❑ ❑ ❑ ❑ ❑ ❑ Very willing to take risks

What about your health?

Not at all willing to take risks ❑ ❑ ❑ ❑ ❑ ❑ ❑ ❑ ❑ ❑ ❑ Very willing to take risks

What about trusting strangers?

Not at all willing to take risks ❑ ❑ ❑ ❑ ❑ ❑ ❑ ❑ ❑ ❑ ❑ Very willing to take risks

Another question about risk taking: Please think about what you would do in the following situation: Imagine that you win 100,000 euros in a lottery. Immediately after receiving the winnings, you receive an offer from a reputable bank for a financial investment that includes the following: There is a chance to double the money within two years. However, there is also an equally high risk of losing half of the staked money. You can invest all or part of the money in the following way or reject the offer.
Which part of the lottery winnings would you use for the on the one hand risky, on the other hand profit promising investment?

❑ the entire amount of 100,000 euros

❑ the amount of 80,000 euros

❑ the amount of 60,000 euros

❑ the amount of 40,000 euros

❑ the amount of 20,000 euros

❑ nothing at all

Have you donated to good causes in the last year?

- ❑ Yes
- ❑ No

Do you have family members who are practicing as physicians?

- ❑ Yes
- ❑ No

Do you have family members who are employed at the hospital?

- ❑ Yes
- ❑ No

Have you had a hospital stay in the last year?

- ❑ Yes
- ❑ No

In social situations, I'm usually the one who makes the first move.

Does not apply at all ❑ ❑ ❑ ❑ ❑ Fully applies

People see me as hard-hearted person.

Does not apply at all ❑ ❑ ❑ ❑ ❑ Fully applies

I try to give generously to those in need.

Does not apply at all ❑ ❑ ❑ ❑ ❑ Fully applies

People sometimes tell me that I'm too stubborn.

Does not apply at all ❑ ❑ ❑ ❑ ❑ Fully applies

It wouldn't bother me to harm someone I didn't like.

Does not apply at all ❑ ❑ ❑ ❑ ❑ Fully applies

I rarely express my opinions in group meetings.

Does not apply at all ❑ ❑ ❑ ❑ ❑ Fully applies

I have sympathy for people who are less fortunate than I am.

Does not apply at all ❑ ❑ ❑ ❑ ❑ Fully applies

I find it hard to compromise with people when I really think I'm right.

Does not apply at all ❑ ❑ ❑ ❑ ❑ Fully applies

A.2.4 Screenshots of the Comprehension Questions for the Individual Condition

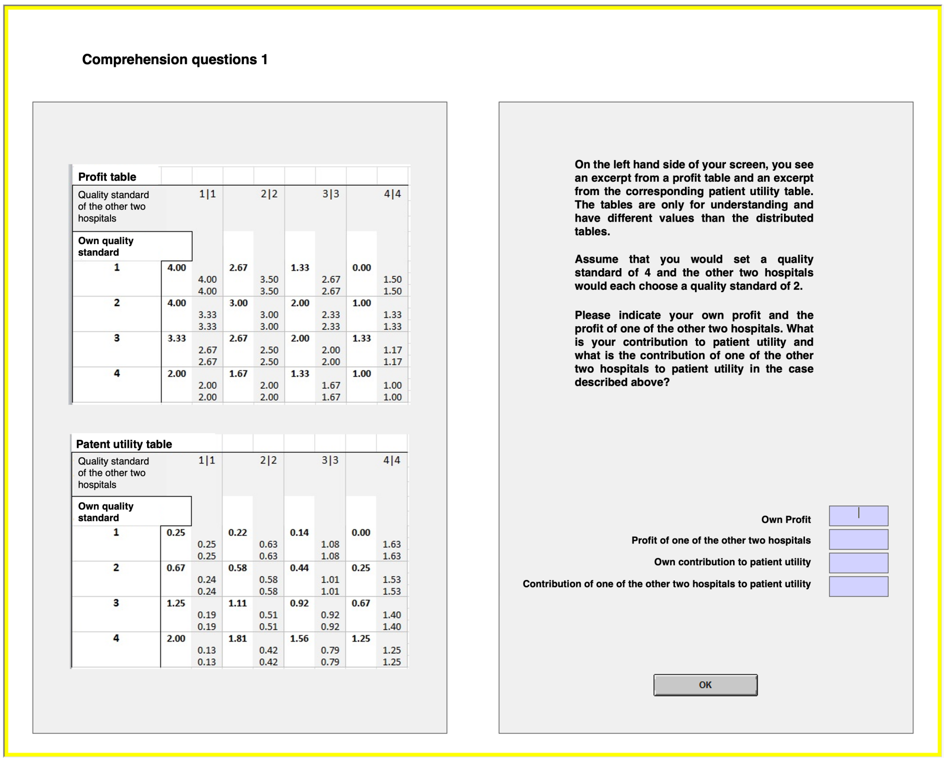

Comprehension questions 1

Profit table

Own quality standard \ Quality standard of the other two hospitals	1|1	2|2	3|3	4|4
1	**4.00**<br>4.00<br>4.00	**2.67**<br>3.50<br>3.50	**1.33**<br>2.67<br>2.67	**0.00**<br>1.50<br>1.50
2	**4.00**<br>3.33<br>3.33	**3.00**<br>3.00<br>3.00	**2.00**<br>2.33<br>2.33	**1.00**<br>1.33<br>1.33
3	**3.33**<br>2.67<br>2.67	**2.67**<br>2.50<br>2.50	**2.00**<br>2.00<br>2.00	**1.33**<br>1.17<br>1.17
4	**2.00**<br>2.00<br>2.00	**1.67**<br>2.00<br>2.00	**1.33**<br>1.67<br>1.67	**1.00**<br>1.00<br>1.00

Patent utility table

Own quality standard \ Quality standard of the other two hospitals	1|1	2|2	3|3	4|4
1	**0.25**<br>0.25<br>0.25	**0.22**<br>0.63<br>0.63	**0.14**<br>1.08<br>1.08	**0.00**<br>1.63<br>1.63
2	**0.67**<br>0.24<br>0.24	**0.58**<br>0.58<br>0.58	**0.44**<br>1.01<br>1.01	**0.25**<br>1.53<br>1.53
3	**1.25**<br>0.19<br>0.19	**1.11**<br>0.51<br>0.51	**0.92**<br>0.92<br>0.92	**0.67**<br>1.40<br>1.40
4	**2.00**<br>0.13<br>0.13	**1.81**<br>0.42<br>0.42	**1.56**<br>0.79<br>0.79	**1.25**<br>1.25<br>1.25

On the left hand side of your screen, you see an excerpt from a profit table and an excerpt from the corresponding patient utility table. The tables are only for understanding and have different values than the distributed tables.

Assume that you would set a quality standard of 4 and the other two hospitals would each choose a quality standard of 2.

Please indicate your own profit and the profit of one of the other two hospitals. What is your contribution to patient utility and what is the contribution of one of the other two hospitals to patient utility in the case described above?

Own Profit

Profit of one of the other two hospitals

Own contribution to patient utility

Contribution of one of the other two hospitals to patient utility

OK

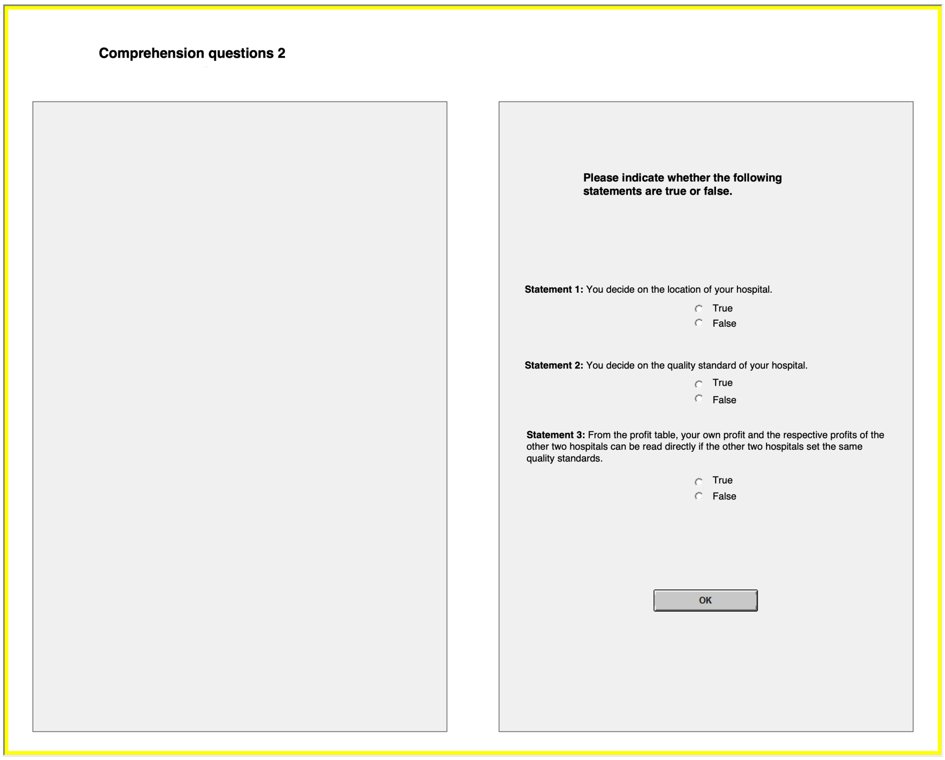

Comprehension questions 2

Please indicate whether the following statements are true or false.

Statement 1: You decide on the location of your hospital.

True
False

Statement 2: You decide on the quality standard of your hospital.

True
False

Statement 3: From the profit table, your own profit and the respective profits of the other two hospitals can be read directly if the other two hospitals set the same quality standards.

True
False

OK

A.3 Model with Altruism

The optimal quality decision for hospital i can be found by optimizing for q_i:

$$\frac{\partial \Omega_\mathrm{i}}{\partial q_i} = -c \cdot D_i + (p - c \cdot q_i) \cdot \frac{\partial D_i}{\partial q_i} + \alpha \frac{\partial B_i}{\partial q_i} = 0. \tag{8}$$

We can then derive the best response function:

$$q_i(q_j) = \frac{12\,p + 6\,c\,\sum_{j \neq i} q_j - 4\,c\,t + \alpha\left(-3\,\sum_{j \neq i} q_j + 2\,t + 12\,v\right)}{6\,(4\,c - 3\,\alpha)}. \tag{9}$$

The strategic relationship is given by:

$$\frac{\partial q_i(q_j)}{\partial q_j} = \frac{-\alpha + 2c}{2(4c - 3\alpha)} > 0 \Leftrightarrow \ \alpha > 2c; \alpha < \frac{4c}{3} \tag{10a}$$

$$\frac{\partial q_i(q_j)}{\partial q_j} = \frac{-\alpha + 2c}{2(4c - 3\alpha)} < 0 \Leftrightarrow \ \frac{4c}{3} < \alpha < 2c\,. \tag{10b}$$

While the best response function in Eq. 9 allows quality to be a strategic complement as well as a strategic substitute, semi-altruistic hospitals that maximize their objective functions should treat quality as strategic complement since the second order condition requires $\alpha < \frac{4c}{3}$.

The Nash equilibrium quality $q_{i\alpha}^*$ and profit level $\pi_{i\alpha}^*$ of hospital i is then given by:

$$q_{i\alpha}^* = \frac{6\,(p + \alpha\,v) - t(2\,c - \alpha)}{6\,(c - \alpha)} \quad \text{and} \quad \pi_{i\alpha}^* = \frac{c\,t}{9} - \alpha\frac{t}{12}. \tag{11}$$

In case with altruism levels that do not offset costs, the Nash Equilibrium quality with semi-altruistic hospitals is higher than the Nash Equilibrium quality with pure profit maximizing hospitals if $6\left(v + \frac{p}{c}\right) > t$ holds. Since we assume that travel costs are sufficiently small, i.e. $t < 3\frac{p}{c}$ holds and v, p, and c are non-negative for our parametrization, it can be shown that $6\left(v + \frac{p}{c}\right) > 3\frac{p}{c} > t$ holds. Thus Nash Equilibrium quality with semi-altruistic hospitals is higher than the Nash Equilibrium quality with pure profit maximizing hospitals (i.e. $q_{i\alpha}^* > q_i^*$) and profits are lower compared to pure profit-maximizing hospitals (i.e. $\pi_{i\alpha}^* < \pi_i^*$). In case of altruism levels that offset costs, exists no possible solution for our parametrization.

A.4 Additional Individual Characteristics

In addition to demographic characteristics the questionnaire that was assessed after the experiment also includes questions regarding donation behavior, whether a family member is a physician or is working in a hospital as well as whether the student was hospitalized in the previous year. Moreover, we assessed personality traits according to social boldness, flexibility and altruism by the HEXACO questions (Ashton and Lee, 2009). Patience and general risk attitudes as well as risk attitudes regarding money, health, and strangers are assessed by 10 point Likert scales with higher values associated with higher patience and more risk seeking, respectively (Dohmen et al., 2011). We calculated means of the instrument assuming cardinal rather than ordinal scales. Furthermore, risk attitudes are also assessed by one monetary lottery question. Participants are asked to share an amount of money to a uncertain lottery. Higher stakes assigned to the lottery imply more risk seeking.

Form Table A.1, we can infer that similar to the demographic data, individuals in the individual market condition and in the team market condition are comparable with respect to most of the characteristics. Individuals in the individual market condition seem to have more frequently family members working in a hospital, seem to be less altruistic according to the Hexaco altruism score and are more risk-averse regarding strangers at a significance level of 5%. The differences are prone to multiple testing and are rather small in absolute values. Since students are randomly assigned to the conditions, we do not expect systematic biases by these differences.

Table A.1: Additional Individual Characteristics

	Total (N = 555)	Individual markets (N = 213)	Team markets (N = 342)	p-value
Donation previous year (1 = yes), N (%)	236 (42.52%)	91 (42.72%)	145 (42.40%)	0.9400 a)
Family member is physician (1 = yes), N (%)	79 (14.23%)	32 (15.02%)	47 (13.74%)	0.6750 a)
Family member working in hospital (1 = yes), N (%)	171 (30.81%)	76 (35.68%)	95 (27.78%)	0.0500 a)
Hospitalization previous year (1 = yes), N (%)	91 (16.40%)	32 (15.02%)	59 (17.25%)	0.4910 a)
Hexaco - Social Boldness, mean (Std. Dev.)	3.62 (0.86)	3.58 (0.91)	3.64 (0.83)	0.6739 b)
Haxoco – Flexibility, mean (Std. Dev.)	2.48 (0.87)	2.48 (0.88)	2.48 (0.86)	0.9960 b)
Hexaco – Altruism, mean (Std. Dev.)	3.59 (0.68)	3.51 (0.69)	3.65 (0.67)	0.0197 b)
Patience, mean (Std. Dev.) (higher values = more patient)	5.29 (2.64)	5.27 (2.67)	5.30 (2.62)	0.9244 b)
Risk attitudes, mean (Std. Dev.) (higher values = risk seeking)	4.95 (2.32)	4.94 (2.33)	4.96 (2.32)	0.9687 b)
Risk attitudes regarding money, mean (Std. Dev.) (higher values = risk seeking)	3.12 (2.49)	3.16 (2.55)	3.10 (2.46)	0.9499 b)
Risk attitudes regarding health, mean (Std. Dev.) (higher values = risk seeking)	4.03 (2.68)	4.10 (2.59)	3.98 (2.74)	0.5535 b)
Risk attitudes regarding strangers, mean (Std. Dev.) (higher values = risk seeking)	4.59 (2.48)	4.32 (2.40)	4.75 (2.51)	0.0375 b)
Risk attitudes - lottery				
stake of 0€	171 (30.81%)	62 (29.11%)	109 (31.87%)	0.3690 c)
stake of 20,000€	164 (29.55%)	65 (30.52%)	99 (28.95%)	
stake of 40,000€	128 (23.06%)	58 (27.23%)	70 (20.47%)	
stake of 60,000€	57 (10.27%)	18 (8.45%)	39 (11.40%)	
stake of 80,000€	20 (3.60%)	6 (2.82%)	14 (4.09%)	
stake of 100,000€	15 (2.70%)	4 (1.88%)	11 (3.22%)	

Note:. a) Chi^2-Test b) MWU-Test c) Fisher's Exact-Test.

A.5 Individual characteristics and preferences regarding quality choices

To study the interaction between individual characteristics and quality choices we focus on the first decision made in the experiment, i.e. quality choice in period 1 for individual markets and first quality suggestion in the first majority voting process in period 1 for team markets. The first decision is unaffected by any quality choice of other participants and, thus, should reflect individual preferences best.

Table A.2 shows the regression results for individual markets for different specifications. Older participants set significantly lower qualities in the first period (only specification (2)) at a 5% significance level. Regarding fields of studies, people studying engineering sciences choose higher qualities than economic students (specification (1)). Using economic students as binary variable, economic students choose lower qualities in comparison to students who do not study economics (only specification (2)). Furthermore, participants who score on higher social boldness choose higher quality levels. Other personality traits do not affect quality significantly at a 5% significance level.

The OLS regression results regarding the first quality suggestion in the majority voting process of team markets are presented in Table A.3. The results show that medicine students choose quality levels significantly below quality levels of economic students at a 1% significance level (specification (1)). Furthermore, individuals with a family member who worked in a hospital choose significantly higher quality levels at a 1% significance level.

Table A.2: Regression Results on First Choice of Hospital's Quality Level (Individual Markets)

	(1)	(2)	(3)
Female (1 = yes)	0.609	0.498	0.385
	(0.496)	(0.469)	(0.478)
Age	-0.112	-0.126*	-0.123
	(0.066)	(0.062)	(0.062)
Students			
Economics (reference category)			
Medicine	1.137		
	(0.935)		
Healthcare management	1.077		
	(1.908)		
Educational sciences	0.772		
	(0.782)		
Humanities	0.789		
	(0.689)		
Natural sciences	1.026		
	(0.674)		
Engineering sciences	2.138*		
	(0.882)		
Other studies	0.026		
	(0.871)		
Economic student (1 = yes)		-0.975*	-0.895
		(0.479)	(0.488)
Hexaco - Social Boldness	0.564*	0.605*	0.567*
	(0.254)	(0.248)	(0.243)
Haxoco - Flexibility	0.113	0.128	0.205
	(0.271)	(0.268)	(0.274)
Hexaco - Altruism	0.307	0.234	0.304
	(0.368)	(0.359)	(0.366)
Patience	-0.104	-0.094	-0.091
	(0.0896)	(0.0877)	(0.0878)
Risk attitudes	-0.150	-0.174	
	(0.104)	(0.100)	
Risk attitudes regarding money			-0.132
			(0.098)
Risk attitudes regarding health			0.001
			(0.092)
Risk attitudes regarding strangers			-0.137
			(0.099)
Donation previous year (1 = yes)	0.160	0.254	0.191
	(0.474)	(0.462)	(0.458)
Family member is physician (1 = yes)	-0.280	-0.200	-0.245
	(0.689)	(0.667)	(0.677)
Family member working in hospital (1 = yes)	0.151	0.129	0.238
	(0.513)	(0.504)	(0.506)
Hospitalization previous year (1 = yes)	-0.445	-0.363	-0.456
	(0.631)	(0.618)	(0.618)
Constant	8.540***	9.968***	9.750***
	(2.152)	(2.101)	(2.148)
N	213	213	213
adjusted R^2	0.048	0.060	0.057

Notes: OLS regression. Standard errors in parentheses. * $p < 0.05$, ** $p < 0.01$, *** $p < 0.001$.

Table A.3: Regression Results on First Quality Choice in Majority Process (Team Markets)

	(1)	(2)	(3)
Female (1 = yes)	0.688	0.652	0.664
	(0.353)	(0.349)	(0.357)
Age	-0.039	-0.041	-0.042
	(0.051)	(0.051)	(0.051)
Students			
Economics (reference category)			
Medicine	-2.941**		
	(0.979)		
Healthcare management	-0.533		
	(1.703)		
Educational sciences	-0.061		
	(0.547)		
Humanities	0.537		
	(0.459)		
Natural sciences	0.027		
	(0.527)		
Engineering sciences	0.221		
	(0.631)		
Other studies	-0.164		
	(0.637)		
Economic student (1 = yes)		-0.035	-0.102
		(0.341)	(0.352)
Hexaco - Social Boldness	-0.094	-0.095	-0.055
	(0.201)	(0.202)	(0.199)
Haxoco – Flexibility	0.146	0.139	0.108
	(0.192)	(0.193)	(0.195)
Hexaco – Altruism	0.420	0.387	0.376
	(0.266)	(0.268)	(0.275)
Patience	0.047	0.067	0.050
	(0.063)	(0.064)	(0.064)
Risk attitudes	0.110	0.117	
	(0.073)	(0.073)	
Risk attitudes regarding money			0.085
			(0.070)
Risk attitudes regarding health			-0.002
			(0.064)
Risk attitudes regarding strangers			0.047
			(0.069)
Donation previous year (1 = yes)	0.283	0.190	0.141
	(0.334)	(0.335)	(0.338)
Family member is physician (1 = yes)	-0.307	-0.380	-0.363
	(0.502)	(0.505)	(0.507)
Family member working in hospital (1 = yes)	1.316***	1.180**	1.216**
	(0.390)	(0.389)	(0.396)
Hospitalization previous year (1 = yes)	-0.368	-0.210	-0.203
	(0.428)	(0.430)	(0.433)
Constant	6.147***	6.297***	6.515***
	(1.689)	(1.746)	(1.782)
N	341	341	341
adjusted R^2	0.066	0.065	0.056

Notes: OLS regression. Standard errors in parentheses. * $p < 0.05$, ** $p < 0.01$, *** $p < 0.001$.

A.6 Robustness Checks Regarding Collusion and Altruism

So far, our analyses show that there is hardly any tendency towards collusion (JPM) on aggregate in both individual and team markets. A way to describe collusion is a joint deviation from the competitive benchmark, which would be 10 in our case, towards the monopoly or JPM outcome (Motta, 2004). We will have a look at two values from this collusive region. First, we take a quality level of 4 or lower (i.e. lower than 5 or 6 which would be the optimal deviation to collusion) as the critical collusion value. We will also look at a quality level of 7 or lower which would be the symmetric counterpart (0.33) for the degree of cooperation to the minimum of -0.33 at the patient optimum.

We can see from Table A.4, that there is not a single market out of 38 in the team conditions that plays collective collusive qualities in one of the 15 periods, neither for a quality threshold of 4 or 7. In the individual conditions, 67 out of 71 markets (more than 90 percent) do not collude collectively over the 15 periods. One market manages to play to collusive periods for 12 periods while four markets collude between 1 and 3 periods. If we relax the quality threshold to 7, still 86 percent of the individual markets do not have a single collusive period.

Table A.4: Number of Markets by Collusive Periods (Quality Level ≤ 4 and ≤ 7)

	Number of collusive periods out of 15 within a market (quality level ≤ 4)					
	0	1	2	3	12	Total
Individual	67 (94.37%)	1 (1.41%)	1 (1.41%)	1 (1.41%)	1 (1.41%)	71
Team	38 (100%)	0	0	0	0	38
	Number of collusive periods out of 15 within a market (quality level ≤ 7)					
Individual	61 (85.92%)	2 (2.82%)	2 (2.82%)	2 (2.82%)	2 (2.82%)	71
Team	38 (100%)	0	0	0	0	38

What could explain the lack of collusion? Did no one initiate a collusion? Maybe the lack of collusion is simply caused by lack of collusion attempts. To answer this question, we look whether at least one hospital set a quality level of 4 or lower in a given period.

Figure A.1 shows the distribution of collusion attempts with a threshold quality level of 4 (grey bars) and 7 (white bars) over 15 periods for the respective markets in individual conditions (left) and team conditions (right). Applying the strict collusion definition of 4, in the

individual condition, around 60 percent of the markets had 0 collusion attempts. In the team conditions, more than 70 percent of markets do not have a single collusion attempt. There are a few more outliers in the individual market with one market with a collusion attempt in every period. Overall, however, the distribution is not significantly different (MWU, p=0.0803). With the relaxed collusion threshold of 7, we observe significantly more collusion attempts in the individual condition compared to the team condition (MWU, p=0.0328).

These observations confirm the idea that the team decision process filters out extreme quality choices. It makes collusion attempts much harder if we take a stricter quality threshold because subjects first have to "convince" the other team members to attempt a collusion. Also, if collusion fails it is likely much harder to convince them to try again. Another explanation might be that in the individual conditions participants only have *rivals* or *competitors* while in the team conditions there is the emphasis on *team*. While the experiment was anonymous, it might be that individuals care more about the opinion or social acceptance by team mates than by rivals and refrain from playing low qualities

Figure A.1: Distribution of Collusion Attempts within 15 periods

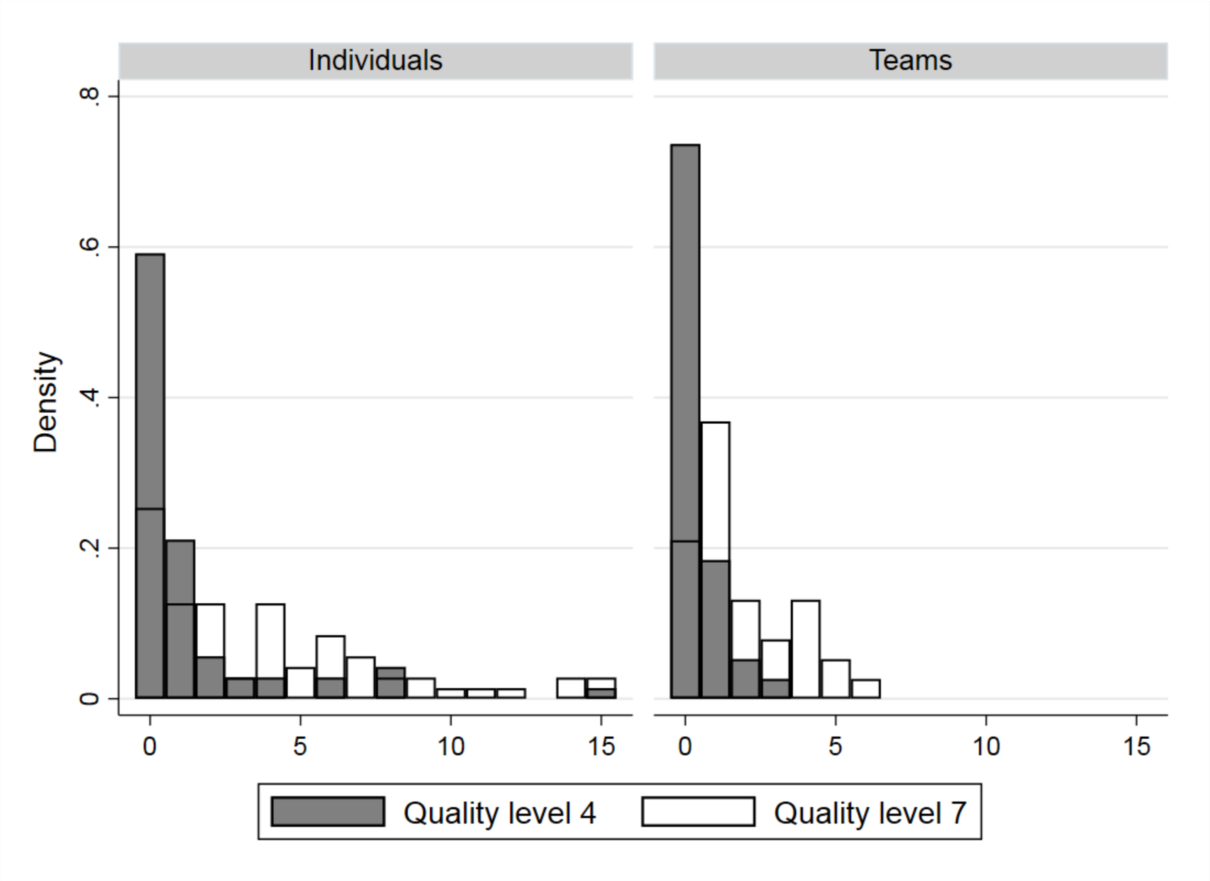

Altruism or Competitiveness

Given that we only find little evidence for collusive behavior on the JPM, we now investigate markets on the other side of the quality spectrum indicating patient centeredness or altruism. The symmetric Nash equilibrium quality is 10. Markets that uniformly play (even) higher qualities might have non-profit-oriented or non-competitive reasons for doing so. While a single occurrence of three simultaneous qualities above 10 might be the outcome of some deviation punishment, consistent plays in this region might explain altruistic behavior.

Similar to our collusion analysis we consider the number of periods where the whole market chose qualities higher than 10. From Figure A.2 we can see that about 25 percent of markets in the individual conditions did not have a single period where all hospitals set a quality higher than 10. In the team conditions only about 10 percent of the markets do not have a unanimously patient oriented period over the whole duration. The difference, however, is not significant (MWU, p=0.2589).

Figure A.2: Number of Periods with Altruistic Quality Choices > 10 in markets (0 individual; 1 team)

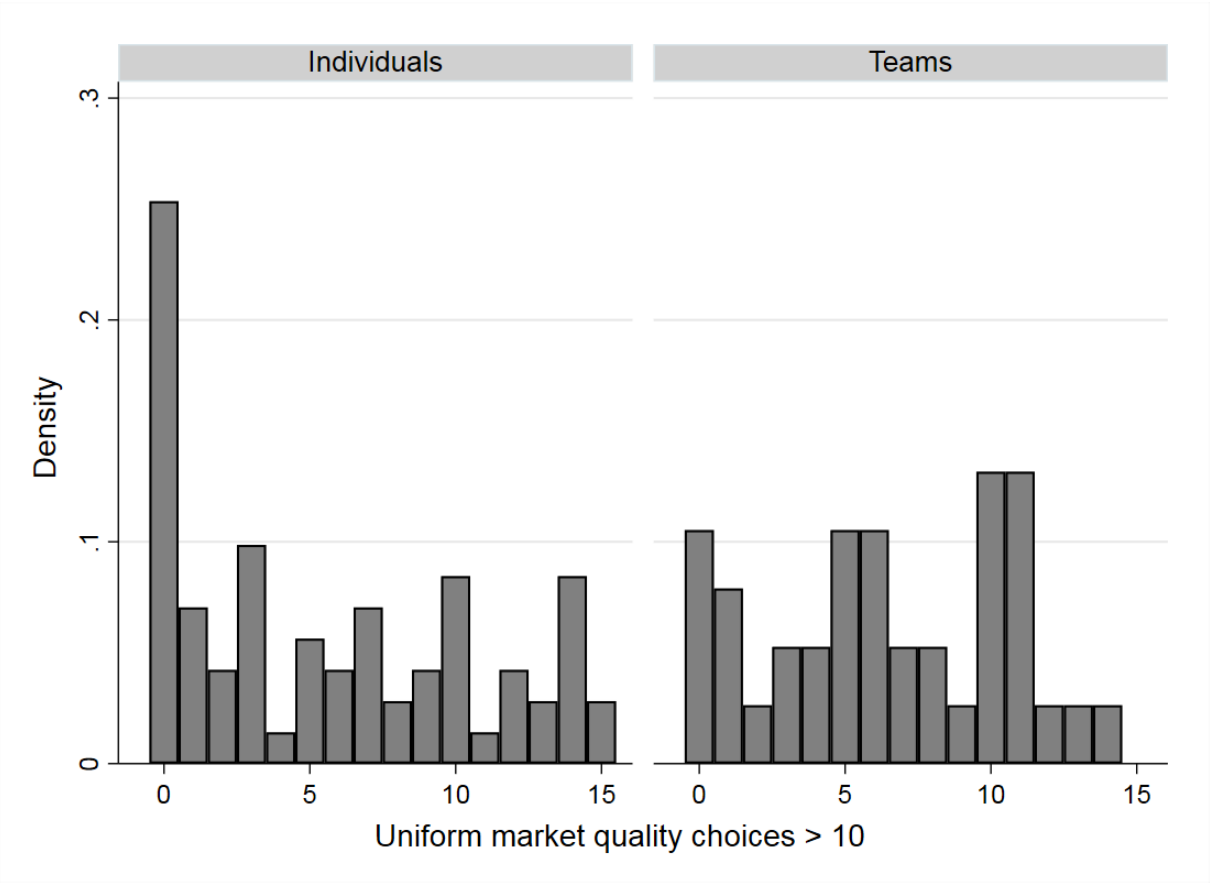

Supplement: Supplementary file 1 — Supplementary file1 (PDF 3350 kb) [file 10754_2024_9366_MOESM1_ESM.pdf]
